# Structure-Based Function Prediction of Uncharacterized Protein Using Binding Sites Comparison

**DOI:** 10.1371/journal.pcbi.1003341

**Published:** 2013-11-14

**Authors:** Janez Konc, Milan Hodošček, Mitja Ogrizek, Joanna Trykowska Konc, Dušanka Janežič

**Affiliations:** 1 National Institute of Chemistry, Ljubljana, Slovenia; 2 University of Primorska, Faculty of Mathematics, Natural Sciences and Information Technologies, Koper, Slovenia; University of Maryland, Baltimore, United States of America

## Abstract

A challenge in structural genomics is prediction of the function of uncharacterized proteins. When proteins cannot be related to other proteins of known activity, identification of function based on sequence or structural homology is impossible and in such cases it would be useful to assess structurally conserved binding sites in connection with the protein's function. In this paper, we propose the function of a protein of unknown activity, the Tm1631 protein from Thermotoga maritima, by comparing its predicted binding site to a library containing thousands of candidate structures. The comparison revealed numerous similarities with nucleotide binding sites including specifically, a DNA-binding site of endonuclease IV. We constructed a model of this Tm1631 protein with a DNA-ligand from the newly found similar binding site using ProBiS, and validated this model by molecular dynamics. The interactions predicted by the Tm1631-DNA model corresponded to those known to be important in endonuclease IV-DNA complex model and the corresponding binding free energies, calculated from these models were in close agreement. We thus propose that Tm1631 is a DNA binding enzyme with endonuclease activity that recognizes DNA lesions in which at least two consecutive nucleotides are unpaired. Our approach is general, and can be applied to any protein of unknown function. It might also be useful to guide experimental determination of function of uncharacterized proteins.

## Author Summary

For a substantial proportion of proteins, their functions are not known since these proteins are not related in sequence to any other known proteins. Binding sites are evolutionarily conserved across very distant protein families, and finding similar binding sites between known and unknown proteins can provide clues as to functions of the unknown proteins. We choose one of the “unknown function” proteins, and found, using a novel strategy of binding site comparison to construct a hypothetical protein-ligand complex, subsequently validated by molecular dynamics that this protein most likely binds and repairs the damaged DNA similar to known DNA-repair enzymes. Our methodology is general and enables one to determine functions of other proteins currently labelled as “unknown function”. We envision that the methodology presented herein, the binding sites comparisons enhanced by molecular dynamics, will stimulate the function prediction of other uncharacterized proteins with structures in the Protein Data Bank and boost experimental functional studies of proteins of unknown functions.

## Introduction

Experimental determination of protein function is the most reliable way to characterize proteins of unknown activity but it is difficult to prioritize functional experiments amongst the many possible functions a protein could perform. To guide experimentalists, a number of computer approaches have been developed for prediction of protein function [1], [2]. Web portals have been created that allow sharing information about protein structures [3], [4]. In spite of these efforts, the gap between proteins with experimentally determined function and those with unknown function is growing [5], [6]. A recent study suggests that more than 40% of known proteins lack any annotation in public databases although many are evolutionarily conserved and probably possess important biological roles [5].

The *TM1631* gene from *Thermotoga maritima* encodes a protein which is a member of a large and widely distributed Duf72 family of domains of unknown function according to Protein family (Pfam) classification [7]. The structure of Tm1631 has been determined by Joint Center for Structural Genomics (PDB: 1vpq), but inferences as to its function are unreliable, because it enjoys little relationship, only about 7% sequence identity, to proteins with diverse known functions. Currently, in 2013, some 3000 proteins of unknown function in the PDB await characterization of their function, and for about one third of these proteins, including Tm1631, there is little hope that their function will be discovered using conventional methods based on sequence or structure homology [6]. A substantial proportion of these proteins, including Tm1631, has no human analogues and may be an important source, for example of new targets for development of antimicrobials [8]. To elucidate their functions, there is a need for methods that go beyond sequence and structure homology and are able to provide testable hypotheses to guide functional experiments.

Because binding sites are usually more evolutionarily conserved structures and more directly linked to function than complete proteins, comparison of protein binding sites to predict function is an attractive alternative to sequence- or structural homology-based methods [2]. Such evolutionarily conserved binding site structures can be found by local structural alignment algorithms that detect similar residue patterns in protein binding sites irrespective of sequence or fold similarity of proteins [9]–[12]. The algorithm, ProBiS (Protein Binding Sites) [11] compares protein binding sites represented as protein graphs in a pairwise fashion using a fast maximum clique algorithm [13] on protein product graphs, and finds sets of residues that are physicochemically and geometrically related. Querying a target binding site, or target protein structure, against a database of template protein structures, ProBiS retrieves proteins with similar binding sites, as defined in this way and from the resulting alignments it calculates degrees of structural conservation for all surface amino acid residues of the target protein. These degrees, mapped to the protein's surface in different colors, show structural evolutionary conservation in the target protein's surface, and predict the location of binding sites as validated on the set of 39 protein structures with known binding sites [11].

**Figure 1:**
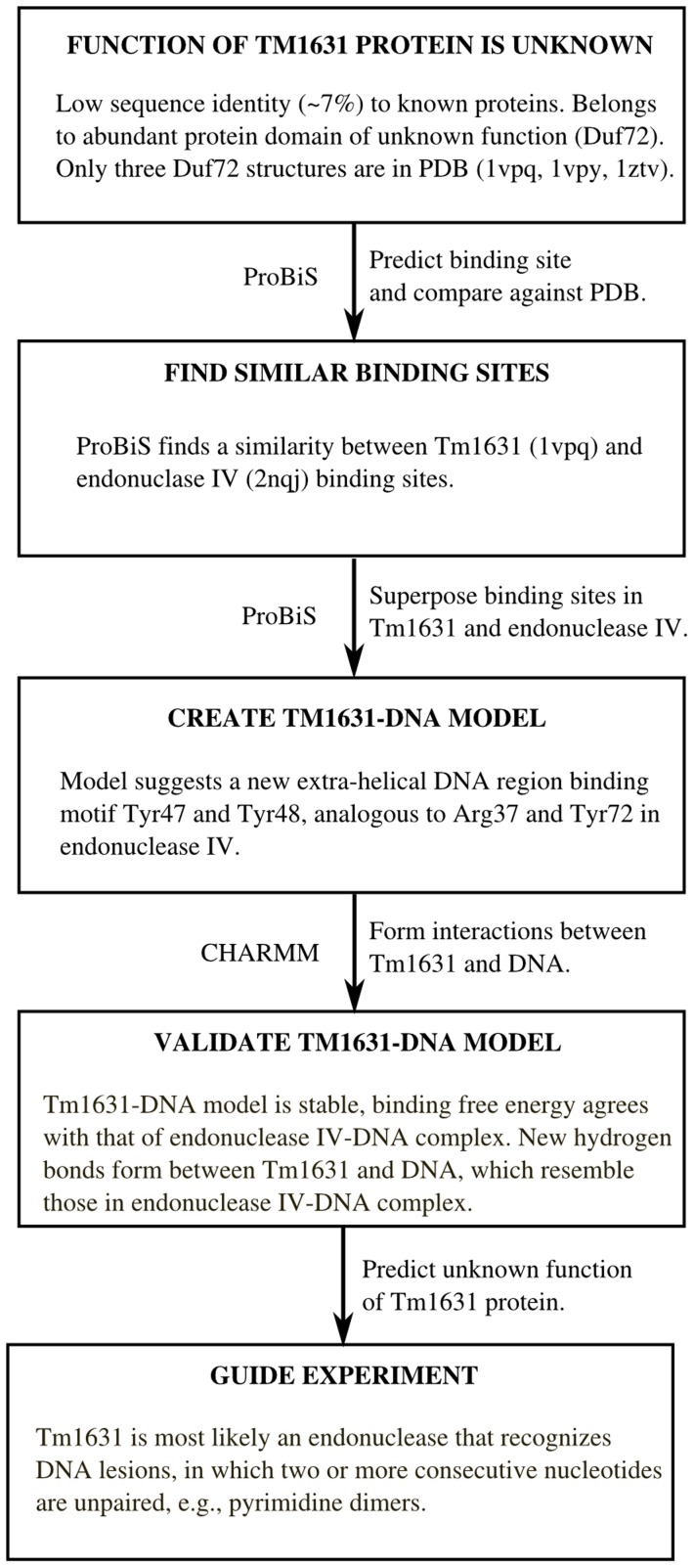
Workflow of the function prediction for the Tm1631 protein structure of unknown function.

In this work, we investigate a new strategy to predict protein function employing ProBiS enhanced by molecular dynamics (MD) simulation (Figure 1), to find structurally evolutionarily conserved binding sites. We validate the new strategy on a set of 369 well-characterized proteins and then apply it to the unknown Tm1631 protein. The strategy proceeds in a number of steps. We first find the binding site on the Tm1631 protein. Then we search for proteins with similar binding sites in the Protein Data Bank [14] (PDB) using the novel binding sites comparison approach described here. In this way, we identify a previously unknown phosphate binding site on Tm1631 that binds a phosphate group of a nucleic acid ligand. To refine the search and narrow down possible functions of Tm1631 protein, we compare this newly identified phosphate binding site with the binding sites in endonuclease IV nucleic acids binding proteins, which are the closest relatives of Tm1631 according to sequence identity, in the α_8_β_8_ triose phosphate isomerase (TIM) barrel fold [15] of which the Tm1631 is a member. A similarity is detected with endonuclease IV DNA-binding site, one of the TIM barrel folds. Based on the superimposition of Tm1631 upon endonuclease IV, we construct a hypothetical model of the Tm1631-DNA complex. Finally, using MD simulation we find that the Tm1631 protein forms favorable interactions with the DNA, which are comparable to those seen in the endonuclease IV-DNA complex. In addition, the binding free energies of Tm1631-DNA model and endonuclease IV-DNA complex are in close agreement. Combined, these findings suggest that the proposed Tm1631-DNA complex is valid, and support speculation that the cleavage of the DNA phosphodiester bond by Tm1631 is distinct from that of endonuclease IV. Tm1631 can thus be identified provisionally as a DNA binding enzyme with endonuclease activity, and experimental investigations can be directed towards the repair of DNA lesions in which at least two consecutive nucleotides in each DNA strand are unpaired, e.g., pyrimidine dimers formed from thymine or cytosine bases in DNA via photochemical reactions [16]. Such comparison of binding sites and generation of hypothetical protein-ligand models followed by molecular dynamics analysis is a method with which function can be assigned to uncharacterized proteins.

## Results

Based on the prediction of its binding site, and comparison of this predicted binding site with the protein structures in the PDB, we propose a DNA-repair function for Tm1631, the protein of unknown activity. We find that despite the low sequence identity of the Tm1631 and endonuclease IV proteins the Tm1631 protein binding site is similar to the known DNA-binding site in endonuclease IV. Construction of a Tm1631-DNA model by superimposition of the similar binding sites found, and running MD simulations shows that Tm1631 enjoys favorable interactions with DNA, similar to those seen in the endonuclease IV-DNA complex. We find that Tm1631 is probably a new endonuclease functioning in a different way than endonuclease IV.

### Detailed view of Tm1631 function

**Figure 2:**
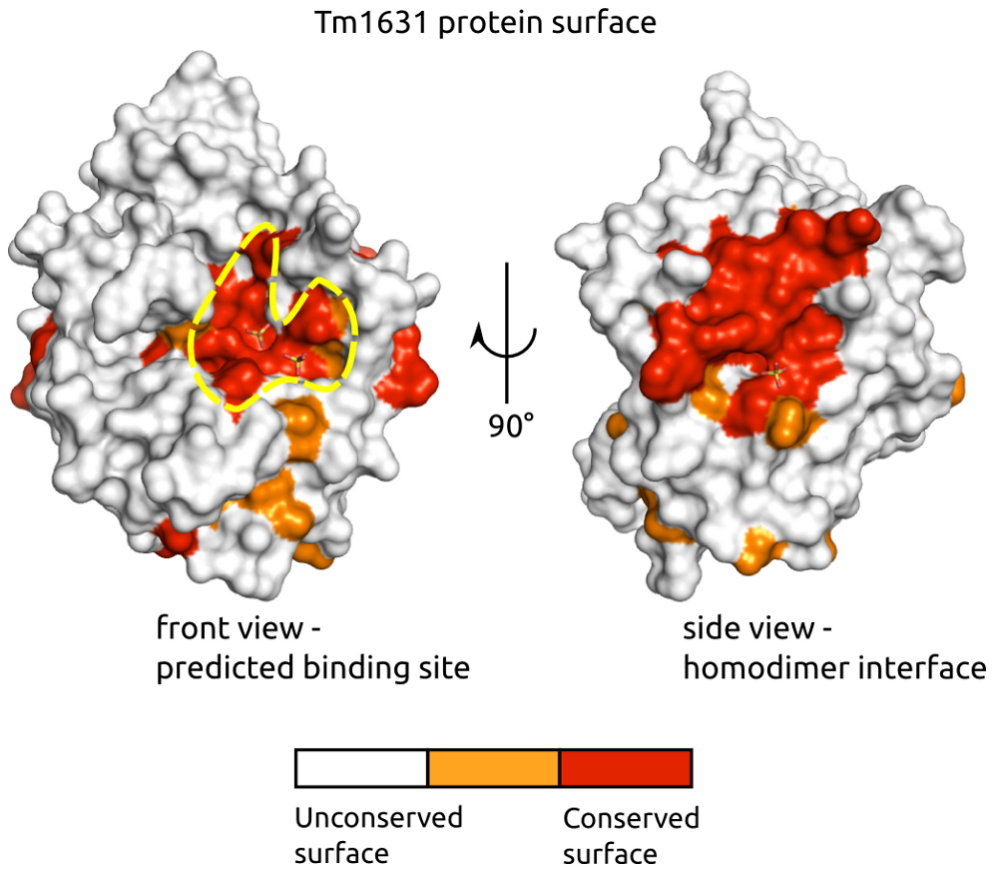
Tm1631 protein surface conservation analysis by ProBiS. Tm1631 is shown in surface representation, which is colored by degrees of structural conservation from unconserved (white) to conserved (red). The predicted binding site is encircled by a yellow dashed line.

Using ProBiS [17], two structurally conserved patches were found on the surface of the Tm1631. The first lies in a groove in the protein surface at the C-terminal side of the TIM barrel (Figure 2, left), and is at a position where proteins of TIM barrel fold often have an active site [18], [19]. We thus considered this patch to be a candidate Tm1631 binding site and used it in a substructure search against the non-redundant PDB. The second structurally conserved patch is on a relatively flat surface (Figure 2, right). Judging by the results from PISA program [20], this second patch is a homodimer binding site on Tm1631. We focused our further investigation on the first patch since it promises to reveal more than the homodimer binding site about the protein's function.

**Figure 3:**
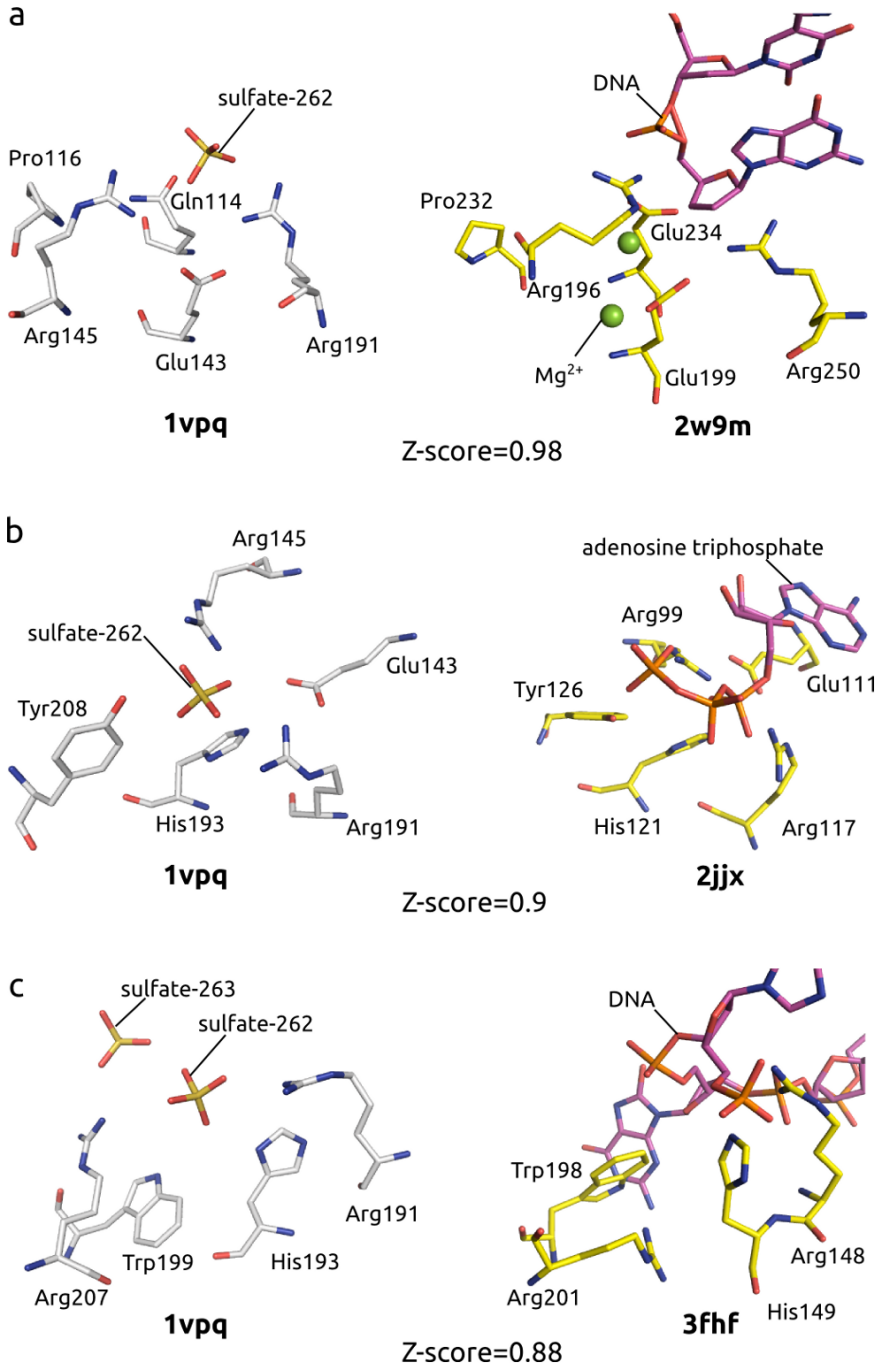
Similar evolutionary patterns in nucleotide binding sites found in PDB using ProBiS. Predicted Tm1631 binding site (left) is similar to (right): (a) active site in DNA binding site of polymerase X (2w9m) with DNA ligand that was transposed from homologous protein structure 3au6; (b) allosteric site in uridine monophosphate kinase (2jjx); (c) active site of DNA-glycosylase (3fhf) with DNA ligand transposed from homologous protein structure 3knt.

We compared the predicted binding site in the Tm1631 protein with protein structures from the non-redundant PDB using binding site comparison approach (see Methods). This comparison showed that the predicted binding site in Tm1631 is very similar to various nucleotide and nucleic acids binding sites in proteins with folds unrelated to the fold of Tm1631 (Table 1 and Table S1 in Text S1). Out of 10 highest ranked similar binding sites, six were DNA or RNA binding sites, and two were nucleotide binding sites. Highest ranked were binding sites in enzymes involved in DNA replication (Figure 3a), transfer of phosphate groups (Figure 3b), and DNA repair (Figure 3c). These results indicated that the predicted binding site in Tm1631 probably binds a nucleotide ligand.

**Table 1:** Top-ranked similar binding sites in proteins of different folds found using the predicted binding site in Tm1631 as query to the binding site comparison approach.^a^

Rank	PDB	Ligand	Function
1	3qrf	DNA	DNA-binding protein
2	2w9m	DNA	DNA replication
3	3zte	RNA	RNA-binding protein
4	2jjx	Nucleotide	Transferase/kinase
5	2vy0	Other	Hydrolase
6	3fhf	DNA	DNA repair
7	1nsc	Other	Hydrolase
8	1lvg	Nucleotide	Transferase/kinase
9	1nio	RNA	Hydrolase
10	3zzs	RNA	Transcription

a The entire list of similar binding sites is in Table S1 in Text S1.

Further, we detected residues predisposed to phosphate binding within the predicted nucleotide binding site. Similarities with the phosphate binding patterns that we found in similar nucleotide binding sites were concentrated on the highly conserved patch of residues near the co-crystallized sulfate ion (sulfate-262) in Tm1631 (Figure 3), suggesting that this surface patch is the phosphate binding site in Tm1631. Uridine monophosphate kinase (2jjx), for example, contains a phosphate binding pattern of residues Tyr/His/Arg/Glu/Arg that almost perfectly matched the Tm1631 residues near the sulfate-262 in their type and orientation (Figure 3b).

The phosphate binding site is most likely also the active site in the Tm1631 protein, as judged from similarity with active sites in polymerase X (2w9m), guanylate kinase (1lvg), and others (Table 1). Based on the reactions performed by the similar active sites found, Tm1631 can act on a phosphate group of a nucleotide catalyzing nucleophilic substitution or phosphoryl transfer. These reactions require electropositive surface potential in the active site that withdraws electrons from the phosphate group, rendering it susceptible to nucleophilic attack [21]. The predicted phosphate binding site in Tm1631 is electropositive (Figure S2 in Text S1), and thus agrees in this respect with the proposed reaction and with the mechanisms operating at the similar active sites found.

The similar binding sites found suggest that the Tm1631 protein is a nucleotide binding enzyme, *i.e.* the identified active site binds and catalyzes a reaction on a nucleotide phosphate group. However, our attempts to construct a model of Tm1631 bound to these ligands were unsuccessful, because the resulting models had too many clashes between the nucleotide ligands and the Tm1631 protein, which prevented further investigation as to how Tm1631 could bind with these ligands.

To find a nucleotide ligand that could bind to the Tm1631, we focused our search for similar binding sites to only TIM barrel proteins that bind nucleotides. According to the standard structural similarity tool [22], endonuclease IV are the most structurally similar nucleotide, specifically, DNA binding proteins out of the TIM barrel proteins, sharing about 7% sequence identity with Tm1631. Using the predicted binding site in Tm1631 as query, we thus searched for similar patterns in all endonuclease IV crystal structures available in the PDB, and found a similar residue pattern in endonuclease IV DNA binding site (PDB: 2nqj, Chain ID: B) (Figure S3 in Text S1). Endonuclease IV is a DNA-repair enzyme that catalyzes phosphodiester bond cleavage in DNA, which is thought to be a nucleophilic substitution reaction on one of the DNA phosphate groups [23], [24]. Endonuclease IV binds to an extra-helical region in DNA, that is a region with interrupted base pairing, and recognizes the apurinic/apyrimidinic (AP) site in the DNA, consisting of a nucleotide lacking a base, but with an intact sugar-phosphate backbone. The enzyme cleaves phosphodiester bond 5′ at the AP site, creating a nick in one of the DNA strands. This was consistent with our findings in fold-unrelated non-redundant PDB proteins, which suggested nucleophilic substitution reaction on phosphate group as the reaction catalyzed by Tm1631. In addition, given the similar residue patterns found within their binding sites, their similar sizes of ∼270 amino acids, and similar electrostatic potential in their binding sites (Figure S2 and S3 in Text S1), implies that the Tm1631 protein could have a related function to endonuclease IV.

### Tm1631-DNA model

**Figure 4:**
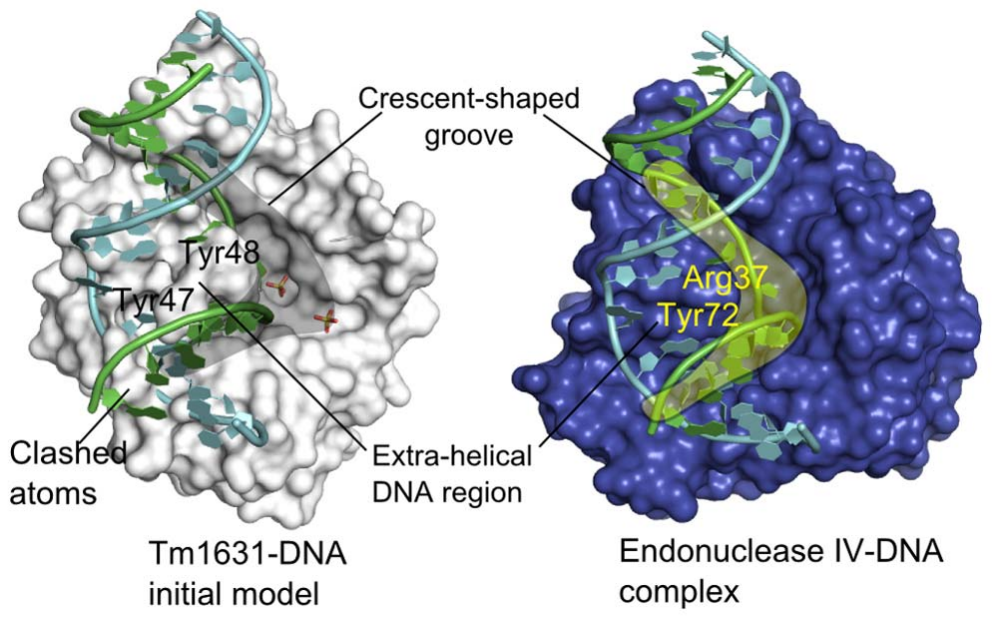
Tm1631-DNA model based on comparison of Tm1631 protein (1vpq) to known endonuclease IV-DNA complex (2nqj) from PDB. Tm1631 is white, endonuclease IV is blue, DNA is green and light-blue cartoons, sulfate ions are CPK sticks, crescent-shaped grooves in both proteins are shaded areas. Initial Tm1631-DNA model; Tyr47 and Tyr48 penetrate the DNA's extra-helical region (left). Endonuclease IV-DNA complex (right).

To test the “endonuclease function” hypothesis, we created a Tm1631-DNA model by transposing the DNA fragment from the endonuclease IV co-crystal structure (2nqj) to the Tm1631 (1vpq) with superimposition of their binding sites. In our model (Figure 4, left), one DNA strand bound into a groove in the surface of Tm1631, so that the reactive phosphate group, *i.e.* the phosphodiester bond 5′ of the AP site that is cleaved by endonuclease IV, was located about 5 Å from the predicted phosphate binding site. There were very few clashes between atoms of the DNA and the Tm1631 in this model and the shape of the groove in the Tm1631 roughly resembled the crescent-shaped DNA-binding groove found in endonuclease IV (Figure 4, right); in both proteins the grooves bound to the same DNA strand. The model suggested that similar to Arg37 and Tyr72 in endonuclease IV, Tyr47 and Tyr48 in Tm1631 bind to the DNA from within the extra-helical region. In endonuclease IV, these residues stack with the DNA bases from within the extra-helical region, and enable the enzyme to distinguish between damaged and normal DNA [23], [24]. Due to their similar physicochemical properties, Tyr47 and Tyr48 could form similar stacking interactions with the bases. The presence of a similar groove in the Tm1631 as can be seen in endonuclease IV, and the two-tyrosine motif that could replace residues binding to the extra-helical region in endonuclease IV, were supportive of our Tm1631-DNA model. However, to view the precise picture of the possible interactions between the Tm1631 and the DNA, we had to refine our model with MD.

### Molecular dynamics simulation of the Tm1631-DNA model

**Figure 5:**
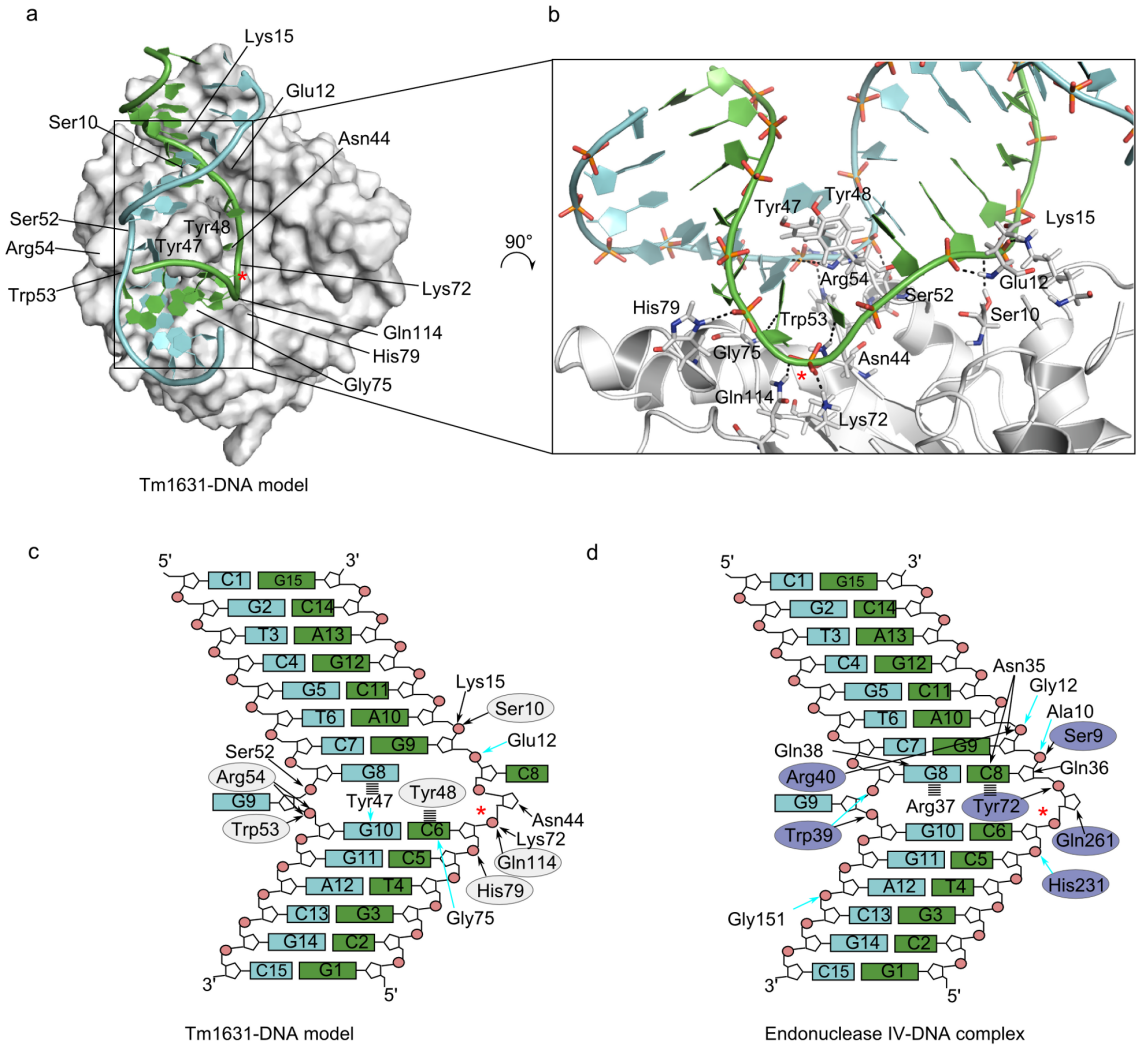
Tm1631-DNA model after 90 ns of MD. Reactive phosphate group in DNA is marked with a red asterisk. (a) Tm1631-DNA model, residues that interact with the DNA are marked. (b) Magnified view of the Tm1631-DNA interface. DNA phosphate groups and residues that interact with the DNA are represented as sticks; black dashed lines denote putative hydrogen bonds and salt bridges. (c) and (d) Schematic picture of Tm1631-DNA and endonuclease IV-DNA interactions. Similar residues in Tm1631 and endonuclease IV binding sites are in white and blue ellipses, respectively. Hydrogen bonds with DNA are shown for amino acid side chains (solid black arrows) and backbone atoms (solid cyan arrows). Stacking interactions with DNA nucleotides are dashed black lines.

To examine the plausibility of the induced fit upon binding of DNA to the Tm1631 we performed an MD simulation of the Tm1631-DNA model in water. Although MD is a theoretical experiment, it showed that DNA fragment remains bound to the Tm1631 throughout the 90 ns of simulation. In addition, new interactions not seen in the initial model formed between Tm1631 and DNA during MD. The final Tm1631-DNA model after MD is shown in Figure 5a and 5b.

We compared the trajectory of Tm1631-DNA model with that of the endonuclease IV-DNA complex, and found many similarities between the Tm1631 and endonuclease IV binding sites that were initially not detected by the similarity detection with ProBiS. Specifically, the residues Ser10, Tyr48, Gln50, Trp53, Arg54, His79, and Gln114 in Tm1631 that hydrogen bonded with the DNA seemed to be direct equivalents, according to their similar positions in the binding site and similar interactions they formed with the DNA, to Ser9, Tyr72, Gln38, Trp39, Arg40, His231, and Gln261 in the known DNA binding site of endonuclease IV (Figure 5c and 5d). Further, we calculated from the trajectories the binding free energy of the Tm1631-DNA model and of the known endonuclease IV-DNA complex; these were −40±14 kcal/mol and −52±23 kcal/mol, respectively (Figure S1 in Text S1). This good agreement of binding free energies indicated that the Tm1631 is a similarly good binder of DNA as endonuclease IV.

The MD also showed that binding of DNA to Tm1631 requires no major structural changes from the either partner. The root-mean-square deviation (RMSD) between the Cα atoms of the Tm1631 before and after MD was ∼1.6 Å and the corresponding RMSD between the phosphorous atoms of DNA was ∼3.7 Å. This last RMSD could also be attributed to the periodical fragmenting and reconstitution of the terminal C14:G17 and G15:C16 base pairs during MD, and to the formation of T-shaped intermediates [25], [26], as well as to the relaxation of atomic clashes between the DNA and Tm1631 during minimization. We also saw occasional unpairing of terminal base pairs in the control simulation of endonuclease IV-DNA complex, which suggests that this is a common process in DNA bound to endonuclease IV. Contrary to the endonuclease IV-DNA simulation, in our Tm1631-DNA model, the C8:G8 base pair opened (Figure 5c), so that the C8 rotated ∼180° to its original position in endonuclease IV (Figure 5d). Most conformational changes in Tm1631 were found in the loop Asn44-Ser52, which binds the extra-helical region of the DNA fragment. The phenyl rings of Tyr47 and Tyr48 rotated ∼100° about χ1 relative to their position in the crystal structure 1vpq to point into the solvent, almost perpendicular to the protein surface, which enabled them to insert themselves through the DNA minor groove, where Tyr47 stacked with G8, and displaced G9 opposite to the AP site; Tyr48 filled the gap left by the missing base of the AP site and stacked with the 5′ base (C6). A simulation of Tm1631 in the unbound state confirmed that these movements also occur without the DNA bound (Figure S4 in Text S1), which indicated that Tyr47 and Tyr48 are in a correct conformation to bind the DNA already in the unbound state of Tm1631. Conformational changes also occurred in the loop Arg195-Asp209 in the Tm1631 but this loop did not bind to the DNA in our model. These last movements could be correlated with the high flexibility of this loop indicated by the high B-factors seen in the crystal structure 1vpq.

## Discussion

We are proposing a structural model of Tm1631 binding to DNA suggesting that Tm1631 could perform a similar DNA repair function as endonuclease IV (Video S1). This model was built by superimposition of binding sites of Tm1631 and endonuclease IV proteins. This superimposition differs from the backbone superimposition obtained with standard structural alignment tool [22], which can only produce a model in which many atoms of the DNA and the Tm1631 clash (Figure S5 in Text S1). In contrast, our model, based on the superimposition of binding sites, had remarkably few clashes between atoms (Figure 4, left).

To validate the binding site comparison approach for function prediction of the Tm1631 protein, we performed an experiment, in which we re-predicted functions of 369 proteins with known functions from the ligAsite [27] benchmark set. We simulated the conditions under which the function of the unknown protein Tm1631 was determined, i.e., proteins of known function with similar sequences were unavailable (for details see Text S1). Our approach correctly predicted 59% of known protein functions in this benchmark set. In contrast, using the BLAST [28] sequence alignment tool instead of the ProBiS [11] algorithm resulted in 43% of protein functions correctly predicted (Table S2 and S3 in Text S1).

The agreement of binding free energies of the Tm1631-DNA model and that of the known endonuclease IV-DNA complex suggests that the hypothetical Tm1631-DNA complex is energetically favorable. This is additionally supported by the similar number of hydrogen bonds formed by the Tm1631 and endonuclease IV with the DNA during MD. In Tm1631-DNA complex there were 12, and in endonuclease IV-DNA complex there were 14 hydrogen bonds (Figure 5c and 5d). This good agreement between the numbers of hydrogen bonds, in addition to the agreement in binding free energies, allows us to posit that the binding affinity of Tm1631 for DNA is similar to that of endonuclease IV.

To validate our Tm1631-DNA model, we also used other computational methods to predict nucleic acid binding site on Tm1631 structure [29], [30], and to search for two-tyrosine motifs in other endonucleases using sequence alignment [28]. We also searched the literature [31] for any information on Duf72 function. The obtained evidence is consistent with our Tm1631-DNA model (Figure S6 and S7 in Text S1).

However, Tm1631 cannot be an endonuclease IV, since the known (PDB: 2x7v) endonuclease IV of *Thermotoga maritima* shares ∼30% sequence identity with other endonucleases IV, whereas the Tm1631 protein has only ∼7% sequence identity with known endonucleases IV. Metal ions have a catalytic role in endonuclease IV, binding with the phosphate 5′ of the AP site and helping cleave the phosphodiester bond. The Tm1631 protein however lacks metal ions in its putative active site, as evidenced in crystal structure 1vpq (and also in homologous structures 1vpy and 1ztv), which additionally distinguishes it from endonuclease IV.

Could therefore the Tm1631 be a new kind of endonuclease that senses a different kind of DNA lesion than endonuclease IV? The two-tyrosine motif in the Tm1631, which prevents base pairing between the two DNA strands, resembles the typical mechanism by which endonucleases sense irregularities like extra-helical region in DNA structure, and this indicates that the Tm1631 could be an endonuclease. However, in Tm1631 the cleavage of the phosphodiester bond must follow a different mechanism than the one employed by endonuclease IV, because, unlike endonuclease IV, Tm1631 has no metal ions in the active site to coordinate the reactive 5′-phosphate of the AP site. Instead, in our Tm1631-DNA model, this phosphate is coordinated by hydrogen bonds from Asn44, Lys72, and Gln114 (Figure 5b). During MD, the phosphate however stays about 3 Å from the predicted phosphate binding site (Figure 3), where it forms additional hydrogen bonds with Arg145 and Arg191 (Figure S8 in Text S1). These hydrogen bonds enable nucleophilic attack on the phosphorous atom by attracting electrons from the phosphorus atom, analogous to catalytic Zn^2+^ ions in endonuclease IV. Metal ions might also be absent due to uncertainties in electron density or experimental conditions, although they actually bind to the Tm1631. A similar binding site found in polymerase X, for example, has magnesium ions (Figure 3c), which supports this hypothesis.

A relatively larger DNA binding groove in Tm1631 compared to the DNA binding groove in endonuclease IV indicates that Tm1631 recognizes a different DNA lesion than endonuclease IV (Figure 4). This would also justify the need for the existence of a new DNA-repair enzyme such as Tm1631 aside from the known endonuclease IV. In Tm1631-DNA model, G8:C8 unpair during MD due to bulky Tyr47 and Tyr48 that require larger extra-helical region than Arg37 and Tyr72 in endonuclease IV (Figure 5a, c). This unpairing of a base pair G8:C8, which is not seen in the endonuclease IV-DNA complex simulation (Figure 5d), suggests that Tm1631 binds preferably DNA lesions, in which two consecutive nucleotides are unpaired, whereas endonuclease IV binds DNA lesions, in which one nucleotide is unpaired, i.e., the AP site. Two consecutive unpaired nucleotides appear for example in pyrimidine dimers DNA lesions, which are result of photodimerization of pyrimidines. Usually, these lesions are repaired by UV endonucleases (see, e.g., 4gle), enzymes related to endonuclease IV [32]. The two-tyrosine motif and larger groove may thus preferentially recognize larger DNA lesions, such as the ones found in pyrimidine dimers.

Finally, we ask, is our developed methodology likely to be useful to those that experimentally determine functions of unknown proteins? We do not have the definitive answer yet. Our model seems to explain well the existing literature data, as well as it agrees with and extends the results of other independent computational methods. The model shows, at the atomic resolution, how the Tm1631 could interact with the DNA. Based on our computational results and good agreement with all available information on this protein structure, we hope that experimentalists will find this problem challenging and will eventually confirm our findings.

## Methods

The protein structure (PDB: 1vpq) encoded by the TM1631 gene was designated here as the query protein. Binding sites were predicted using the ProBiS web server [17] at http://probis.cmm.ki.si. Comparisons of binding site structures were done using the parallel ProBiS program [33] (version 2.4.2) freely available at http://probis.cmm.ki.si/?what=parallel. MD simulations were carried out on the clusters of personal computers (CROW) at the National Institute of Chemistry in Ljubljana [34], using the CHARMM biomolecular simulation program [35] and CHARMMing web server [36]. Structural and dynamic aspects of the molecules were visualized via PyMOL software and surface electrostatics were calculated using APBS program [37].

### Prediction of binding sites on the Tm1631 protein

Using the “Detect Structurally Similar Binding Sites” tool on the ProBiS web server, and selecting the “List of PDB/Chain IDs” option from the “Proteins to Compare Against” drop-down list, the query protein structure 1vpq.A was compared to two crystal structures, 1vpy.A and 1ztv.A; the query protein has about 30% sequence identity with either 1vpy.A or 1ztv.A. From the structural alignments with these two similar proteins, ProBiS calculated the degrees of structural conservation for each residue of the query protein and these were mapped to the surface residues of the query protein to indicate level of evolutionary conservation of each residue. Residues with conservation score of 8–10 on a scale of 1–10, were considered as putative binding site residues [11].

### Binding site comparison

Dynamics simulations of proteins allow study of the flexibility of binding sites at a detailed level. From an MD trajectory, a sequence of snapshots or frames of a protein at different times can be produced [38], [39]. Similarly as improvements in molecular docking [40], using more protein frames as input to a search algorithm such as ProBiS, could increase the likelihood of finding a similar binding site among template protein structures, compared to results obtained with only one static protein frame. Accordingly, we performed a short, 1 ns, MD simulation of the Tm1631 protein (1vpq) in water and quenched 30 frames from this MD trajectory at different time intervals: 20 frames were from the first 100 ps at regular intervals of 5 ps, and 10 frames were from 100 to 1000 ps at intervals of 100 ps. Details of the MD simulation are provided below. Each frame was then separately used as input to the ProBiS program. The region in each frame designated for comparison was defined as the amino acids belonging to the predicted binding site, that is Ser7, Leu43, Glu42, Asn44, Lys72, Gln114, Glu143, Phe144, Arg145, Leu176, Arg191, Trp199, Glu205, Arg207, and Asn239 (Figure 2, left). The selected binding sites in all frames were then compared individually with the entire non-redundant PDB (nr-PDB) of some 31,000 protein structures using the LOCAL and MOTIF options of the ProBiS program, which restrict the search to only the predicted structurally conserved binding site in Tm1631. The nr-PDB is the default database of proteins used by ProBiS; its generation is described elsewhere [11]. The similar substructures that were found in the nr-PDB proteins, were ranked using the Z-Scores assigned by ProBiS, and only those with Z-Score>0.5 were considered further. If different frames shared more similar substructures with the same nr-PDB protein, then the substructure with the highest Z-Score was retained. This procedure resulted in a set of proteins, identified by their PDB IDs and Chain IDs, each having a substructure that was similar to the predicted binding site in Tm1631.

### Filtering of similar binding sites

A similarity between the predicted binding site and a known similar binding site in a different protein is a link that allows determination of the function of the predicted binding site, an uncharacterized region in Tm1631. However, the similar substructures that we found in nr-PDB proteins could occur anywhere on these proteins' surfaces and accordingly we filtered the similar substructures found, so that only those that corresponded with known binding sites remained. The most reliable indication that a region of protein surface is a binding site is if co-crystallized ligands bind to that region in the PDB file of the corresponding protein structure. However, ligands may be absent in a particular protein structure, but can be present in some of the structures of homologous proteins. To define binding sites in the set of newly found similar proteins, we thus superimposed each of these proteins with its >30% sequence identical homologous structures in the PDB, and transposed to the corresponding protein ligands present in the homologous proteins. Modified residues, carbohydrates that are covalently linked to the glycosylation sites of a protein, and non-specific ligands listed at http://www.russelllab.org/wiki/index.php/Non-specific_ligand-protein_binding were not considered to be legitimate ligands. A binding site is defined as residues that are <3 Å away from the ligand atoms. We then filtered the set of similar proteins to obtain only those in which the similar substructure detected by ProBiS corresponded with the known binding site in a template protein. The “similar proteins” that were obtained in this process had binding sites that were similar to the predicted binding site in Tm1631 thus were possible functional analogs of Tm1631.

### Modeling of the Tm1631-DNA complex

We prepared the Tm1631-DNA model using a structural superimposition by ProBiS of crystal structures 1vpq and 2nqj. The model was built with (i) Tm1631 from the crystal structure 1vpq, and (ii) a DNA fragment, in which one nucleotide lacks a base, from the endonuclease IV structure 2nqj. The putative binding site in 1vpq and the known DNA binding site in 2nqj.A were superimposed and the DNA was then transposed from 2nqj.A to 1vpq by copying coordinates of the DNA fragment from 2nqj to the 1vpq crystal structure.

### Molecular dynamics simulations

We performed MD simulation of the Tm1631-DNA model, and two control simulations: first of the unbound Tm1631 protein (PDB: 1vpq), and second of the endonuclease IV-DNA complex (PDB: 2nqj). In the simulation of endonuclease IV-DNA complex, three Zn^2+^ ions were retained in the binding site since they are known to bind to DNA [24]. The control simulations were done for comparison with our model and to determine the flexible regions of the proteins and the DNA. To remove atomic clashes and to optimize the atomic coordinates of the complexes, the steepest descent and adopted basis Newton-Raphson energy minimizations were used. The HBUILD tool in CHARMM was used to add missing hydrogens prior to the minimization. In each case, the DNA ligand was held fixed and the protein was allowed to move freely during the minimization process. The models were then embedded in a cube of water, which was modelled explicitly by a rigid TIP3P model; KCl was added to neutralize the system (for details see Text S1). A trajectory of Tm1631-DNA model, endonuclease IV-DNA complex, and unbound Tm1631 were generated at 310 K and covered 90 ns, 60 ns, and 15 ns, respectively, of MD at constant pressure and temperature employing periodic boundary conditions. In each simulation the first 3 ns of the MD was used for heating (100 ps) and equilibration (2,9 ns); the analysis was performed using the final 20 ns of each simulation, except in the unbound Tm1631 case, where the first 1 ns of simulation was used for binding site comparison. Hydrogen bonds were calculated using the HBOND tool in CHARMM, and only those with occupancy >0.5 were considered. Restraints were used two times during the Tm1631-DNA model simulation to correct the base-pairing in the DNA (Text S1); no restraints were used during last 47 ns to allow the DNA and the Tm1631 protein to position themselves freely responding to physical forces between them.

### Energetics analysis

To compare the relative binding affinities of the Tm1631-DNA and endonuclease IV-DNA complexes, we calculated the relative binding free energies for these complexes using the Molecular Mechanical/Generalized Born Surface Area (MM/GBSA) approach [41], [42]. In this approach, the binding free energy (ΔG_bind_), is calculated as the sum of the changes of the gas phase molecular mechanics energy, ΔE_MM_, the solvation free energy, ΔG_sol_, and the conformational entropy of the system upon binding, −T·ΔS :
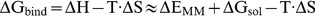
(1)

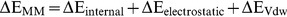
(2)

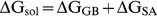
(3)In equation 2, ΔE_MM_ is the sum of ΔE_internal_ (bond, angle, and dihedral energy), ΔE_electrostatic_ (electrostatic energy), and ΔE_Vdw_ (Van der Waals energy); in equation 3, ΔG_sol_ is the sum of electrostatic solvation energy, ΔG_GB_ (polar contribution) and non-electrostatic solvation component, ΔG_SA_ (non-polar contribution). The polar contribution to the desolvation free energy was calculated using the analytical Generalized Born using Molecular Volume (GBMV) model implemented in CHARMM [43], [44], whereas the non-polar energy was estimated by solvent accessible surface area (SASA) calculation implemented within the GB module (Text S1). We assumed that the entropy changes upon binding are similar in both complexes, since in both the DNA is bound in a very similar conformation. Accordingly, to calculate the relative binding free energies, we neglected the entropy term (−T·ΔS). With the exception of the entropy terms, all the energy terms were calculated for 20,000 snapshots sampled at intervals of 1 ps along the last 20 ns of each complex's MD trajectory (Figure S1 in Text S1). We chose the last 20 ns for energy calculation, since in this time interval no new hydrogen bonds formed between the Tm1631 and the DNA.

## Supporting Information

Text S1 Supporting information containing Figure S1–S8, Table S1, further details of MD simulations, electrostatic potential of Tm1631, similar evolutionary pattern in Tm1631 and endonuclease IV, alternative Tm1631-DNA model, validation of Tm1631-DNA model, proposed active site in Tm1631, binding site comparison results, Table S2 and S3, validation of binding site comparison as function prediction approach.
(DOC)

Video S1 A movie illustrating the prediction of Tm1631 protein function.
(MP4)
