# Supplementary material for: Structure-Based Function Prediction of Uncharacterized Protein Using Binding Sites Comparison
Source: PLoS Comput Biol. 2013 Nov 14;9(11):e1003341. doi: 10.1371/journal.pcbi.1003341 (PMC3828134; doi:10.1371/journal.pcbi.1003341)
Supplement: Text S1 — Supporting information containing Figure S1–S8, Table S1, further details of MD simulations, electrostatic potential of Tm1631, similar evolutionary pattern in Tm1631 and endonuclease IV, alternative Tm1631-DNA model, validation of Tm1631-DNA model, proposed active site in Tm1631, binding site comparison results, Table S2 and S3, validation of binding site comparison as function prediction approach. (DOC) [file pcbi.1003341.s001.doc]

Structure-Based Function Prediction of Uncharacterized Protein using Binding Sites Comparison

Supporting Information

Janez Konc,^1^ Milan Hodošček,^1^ Mitja Ogrizek,^1^ Joanna Trykowska Konc,^1^ Dušanka Janežič1,2,[[1]](#footnote-2)*

*^1^National Institute of Chemistry, Ljubljana, Slovenia*

*^2^University of Primorska, Faculty of Mathematics, Natural Sciences and Information Technologies, Koper, Slovenia*

E-mail: dusa@cmm.ki.si

# Molecular dynamics simulations and energetics analysis

We performed a 90 ns molecular dynamics (MD) simulation of Tm1631-DNA model, a 60 ns MD of endonuclease IV-DNA complex, and a 15 ns MD of unbound Tm1631 protein in solvent using the CHARMM program with the all-atom parameter set PARAMALL27 library for proteins and nucleic acids. Cubic water box of dimensions 80×80×80 Å was used in all three simulated systems. In TM1631-DNA model and endonuclease IV-DNA complex simulation, 35 K^+^ and 2 Cl^-^ ions were added to 14962 and 15262 waters, respectively; in unbound Tm1631 simulation, 48 K^+^ and 40 Cl^-^ ions were added to 15169 waters. We used the default protonation states of ionizable residues assigned by the CHARMMing web interface [1]. We used the constant temperature and pressure ensemble. We used the TIP3P water model together with the SHAKE algorithm.

Two times during the Tm1631-DNA model simulation, restraints were applied using RESD command in CHARMM to correct broken base pairs in the DNA. In 21 ns, these restraints were applied for 1 ns with the restraining force (kval) of 1.0 to the base pairs C8:G8, G9:C7, A10:T6, C11:G5, C14:G2, G15:C1, C6:G10, and C5:G11 to correct the distances between their hydrogen bonding atoms to those seen in DNA from crystal structure 2nqj. In 43 ns, similar restraints were applied for 1 ns to the base pairs G9:C7, A10:T6, and C11:G5. These corrections were needed, because in the unbound Tm1631, the extra-helical DNA region binding residues Tyr47 and Tyr48 point as seen in Figure S4 (left), causing clashes with the atoms of the DNA in our initial Tm1631-DNA model; during simulation these strains between the initial conformations of Tyr47 and Tyr48 and the DNA destabilized hydrogen bonds between mentioned DNA base pairs.

To compare the relative binding affinities of the Tm1631-DNA and endonuclease IV-DNA complexes, we calculated the relative binding free energies (∆G_bind_) for these complexes using the Molecular Mechanical/Generalized Born Surface Area (MM/GBSA) approach (Figure S1). We used last 20 ns of each simulation to calculate average ∆G_bind_ of each complex. We set the parameters for the Generalized Born using Molecular Volume (GBMV) model to the following values: TOL (1e-10), MEM (20), CUTA (20), DN(1.0), BUFR (0.2), EPSILON (80), BETA (-12), SHIFT (-0.1), SLOPE (0.9), LAMBDA1 (0.5), P1 (0.45), P2 (1.25), P3 (0.65), P6 (8), ONX (1.9), OFFX (2.1), CORR (1), ALFRQ (1), SON (1.2), SOFF (1.5), FAST (1), SGBFRQ (4), SXD (0.3), WTYP (1), NPHI (5), SA (0.00542), and SB (0.9).


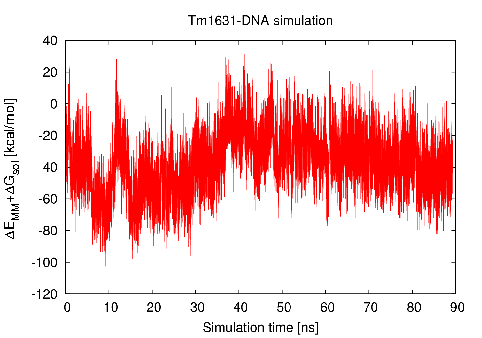

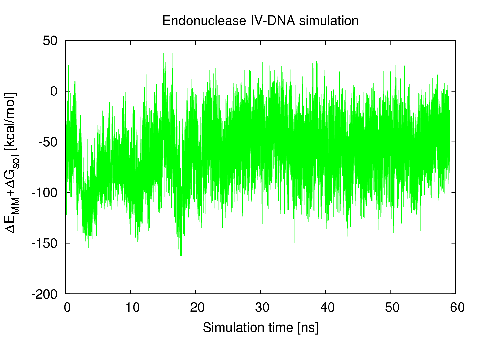
Figure S1**.** Plot of binding enthalpy (∆E_MM_+∆G_sol_) for Tm1631-DNA model over 90 ns of MD trajectory (left), and for endonuclease IV-DNA complex over 60 ns of MD trajectory (right).

# Electrostatic potential in Tm1631

The electrostatic potential of Tm1631 protein surface is shown in Figure S2. The predicted binding site is inside the groove in the center of the protein.


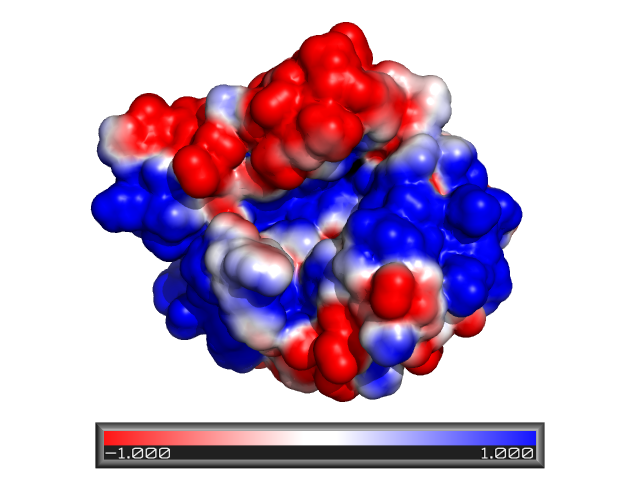


Figure S2. Electrostatic potential mapped to the surface of the Tm1631 protein. Positive surface potential is blue; negative surface potential is red.

# Similar evolutionary pattern in Tm1631 and endonuclease IV

The similar binding sites of the Tm1631 and endonuclease IV found by ProBiS is shown in Figure S3. The ProBiS parameters used were (i) binding site in Tm1631 protein (1vpq.A) to be compared was defined as a 7 Å region around the co-crystallized sulfate-262; (ii) each endonuclease IV protein PDB file was divided into individual protein chains (e.g., 2nqj.B), and entire chains were used in comparison; (iii) LOCAL option in ProBiS was disabled to allow for some flexibility of proteins during the comparison.


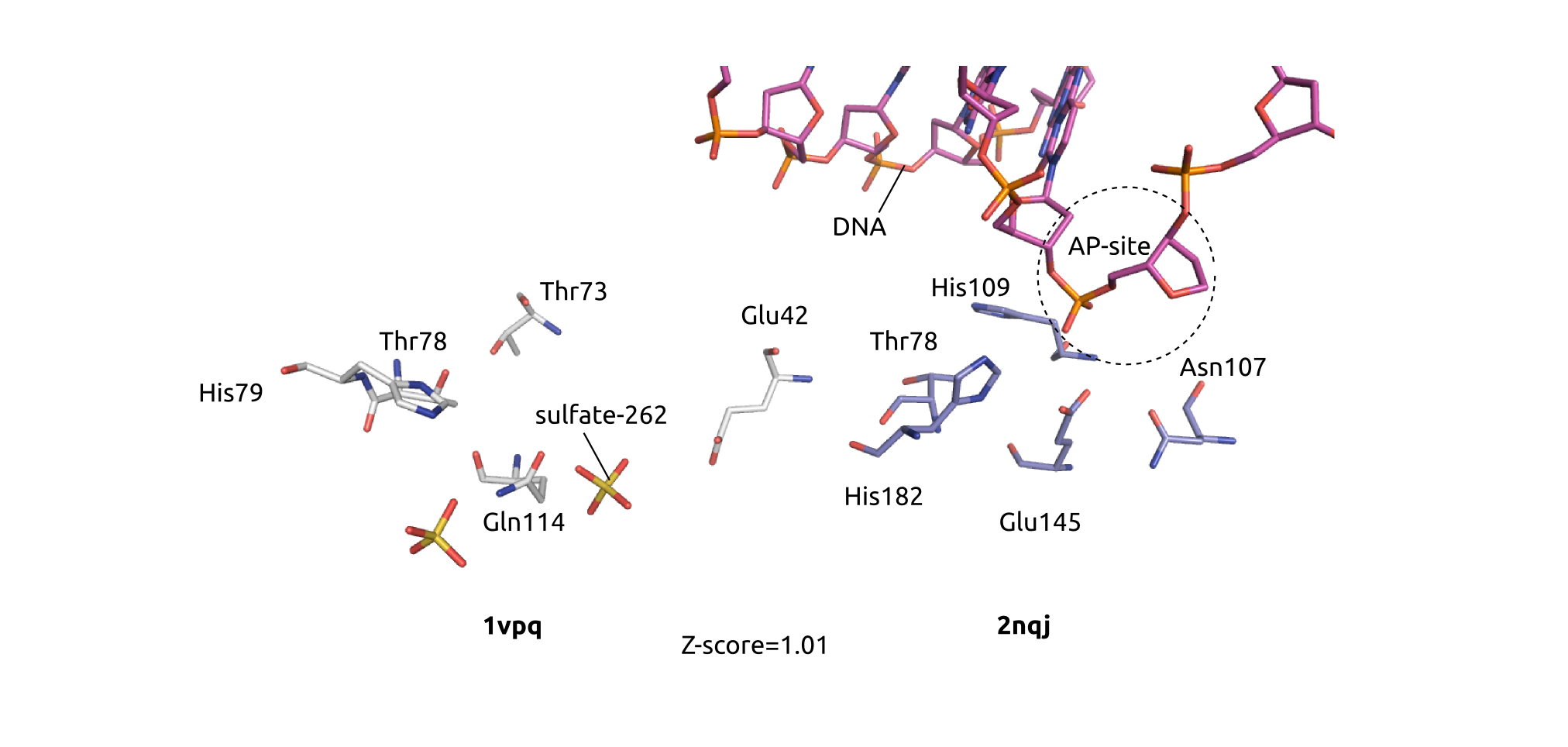


Figure S3. Similar evolutionary pattern in the predicted Tm1631 and endonuclease IV binding sites. Amino acids of Tm1631 from crystal structure 1vpq.A are colored white (left) and in endonuclease IV from 2nqj.B atoms are colored blue (right). The DNA fragment bound (purple) was transposed from chain A to chain B of protein in crystal structure 2nqj.

# Molecular dynamics of unbound Tm1631 protein

A conformational change of Tyr47 and Tyr48 is visible after 7 ns of MD of unbound Tm1631 protein in solvent (Figure S4). This positions the two tyrosines in a conformation similar to that seen in our Tm1631-DNA model, suggesting that the two tyrosines are in a correct conformation for DNA binding already in the unbound Tm1631 protein.


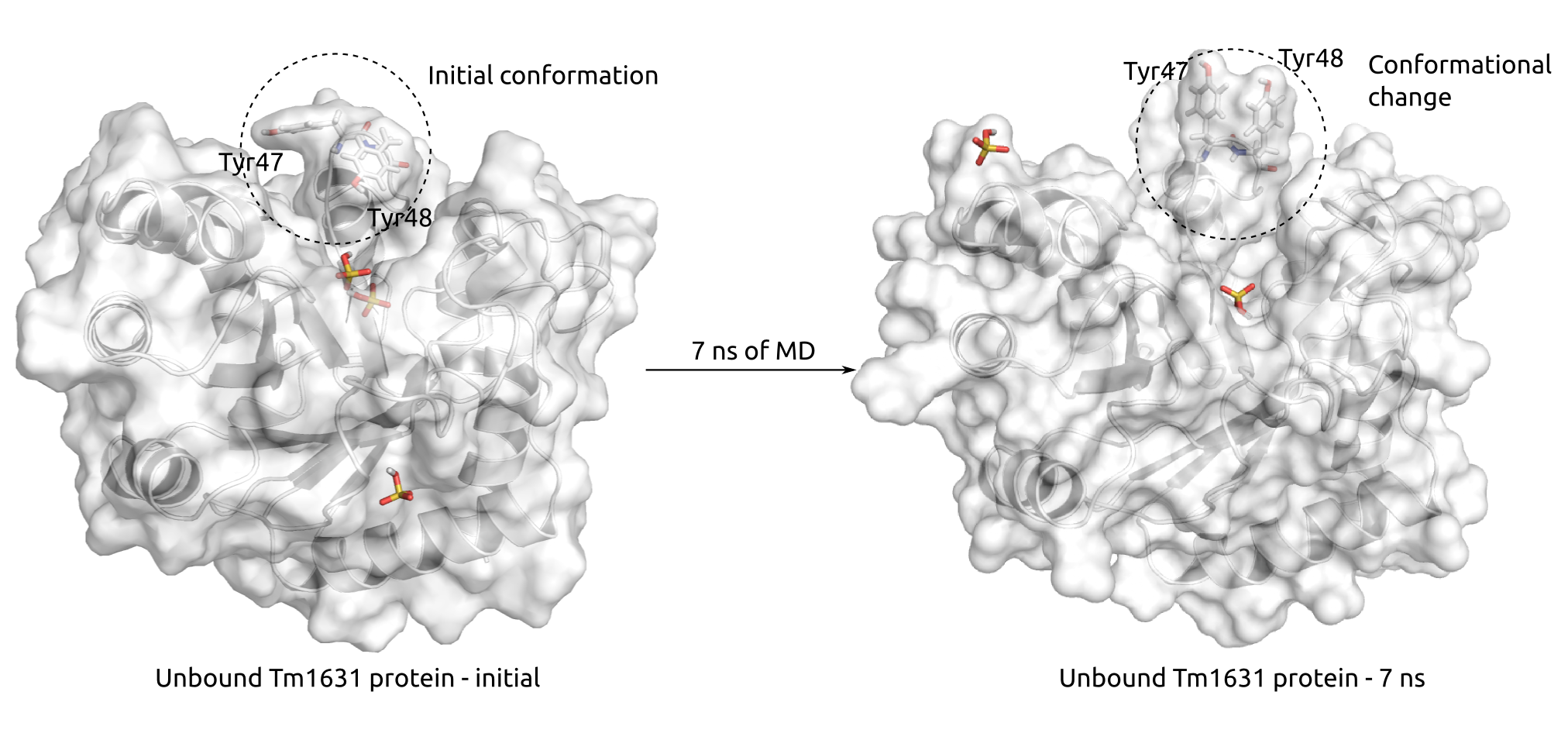


Figure S4. Conformational change in the two-tyrosine motif after 7ns of MD simulation of unbound Tm1631 protein. Tm1631 protein is white transparent surface and cartoon model; Tyr47 and Tyr48 are sticks; sulfate ions are sticks colored using CPK scheme.

# Alternative Tm1631-DNA model

An alternative Tm1631-DNA model is obtained by superimposition with a standard structural alignment tool [2]. In this model, Tyr47 and Tyr48 are not bound to the extra-helical region of the DNA and there are many clashes between atoms of the DNA and the Tm1631 protein (Figure S5). We created a simple program to assess the number of clashed atoms. This program counted the number of clashed protein atoms around each DNA atom. A clash was defined as two atoms whose distance between their centers is less than the sum of their vdW radii. The DNA atoms having >4 clashed protein atoms were considered as severely clashed. Using this metrics, the alternative Tm1631-DNA model obtained using standard structural alignment tool [2] had 41 severely clashed atoms out of the total of 127 clashed atoms, whereas the Tm1631-DNA model obtained using ProBiS had only 27 severely clashed atoms out of 117 clashed atoms.


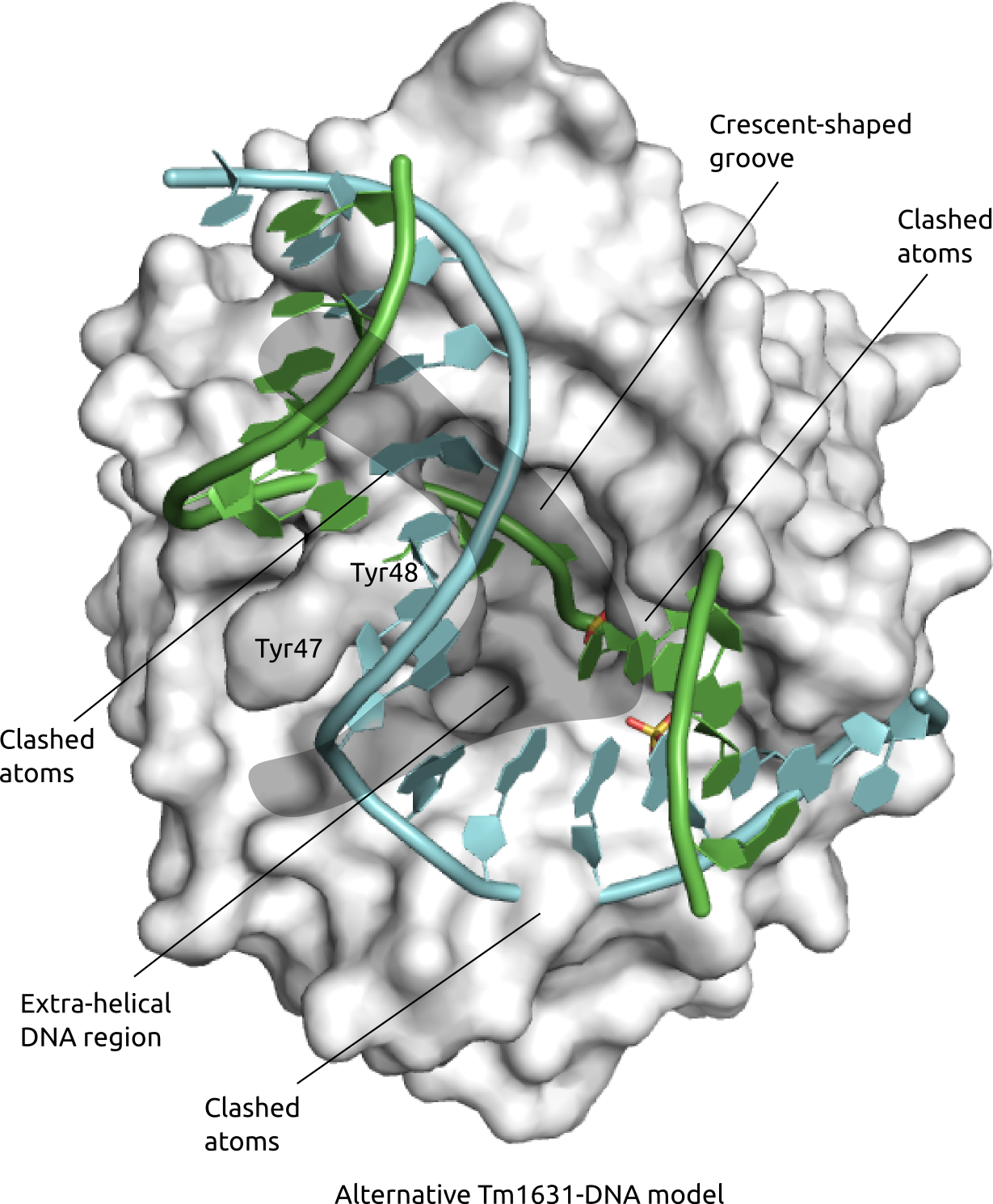


Figure S5. Alternative Tm1631-DNA model obtained using global superimposition of 1vpq and 2nqj crystal structures. Tm1631 protein is white surface; DNA is green and blue cartoons; sulfate ions are sticks models colored using CPK scheme.

# Validation of Tm1631-DNA model

To validate our Tm1631-DNA model, we also used computational methods other than binding sites comparison and MD. We submitted the Tm1631 crystal structure to two servers, DISPLAR [2] and DR-BIND [3], that predict DNA-binding sites using a neural network approach. Their predictions of a DNA binding site on Tm1631 were in excellent agreement with the DNA-binding site postulated by our Tm1631-DNA model (Figure S6). We also searched literature for previous research related to the function of the Duf72 protein family. Dalrymple et al., discovered a sequence motif in proteins of the Duf72 protein family, characteristic of proteins that bind to beta sliding clamp protein and based on this finding, predicted that Duf72 protein family is involved in DNA repair [4,5]. This evidence is consistent with our results.


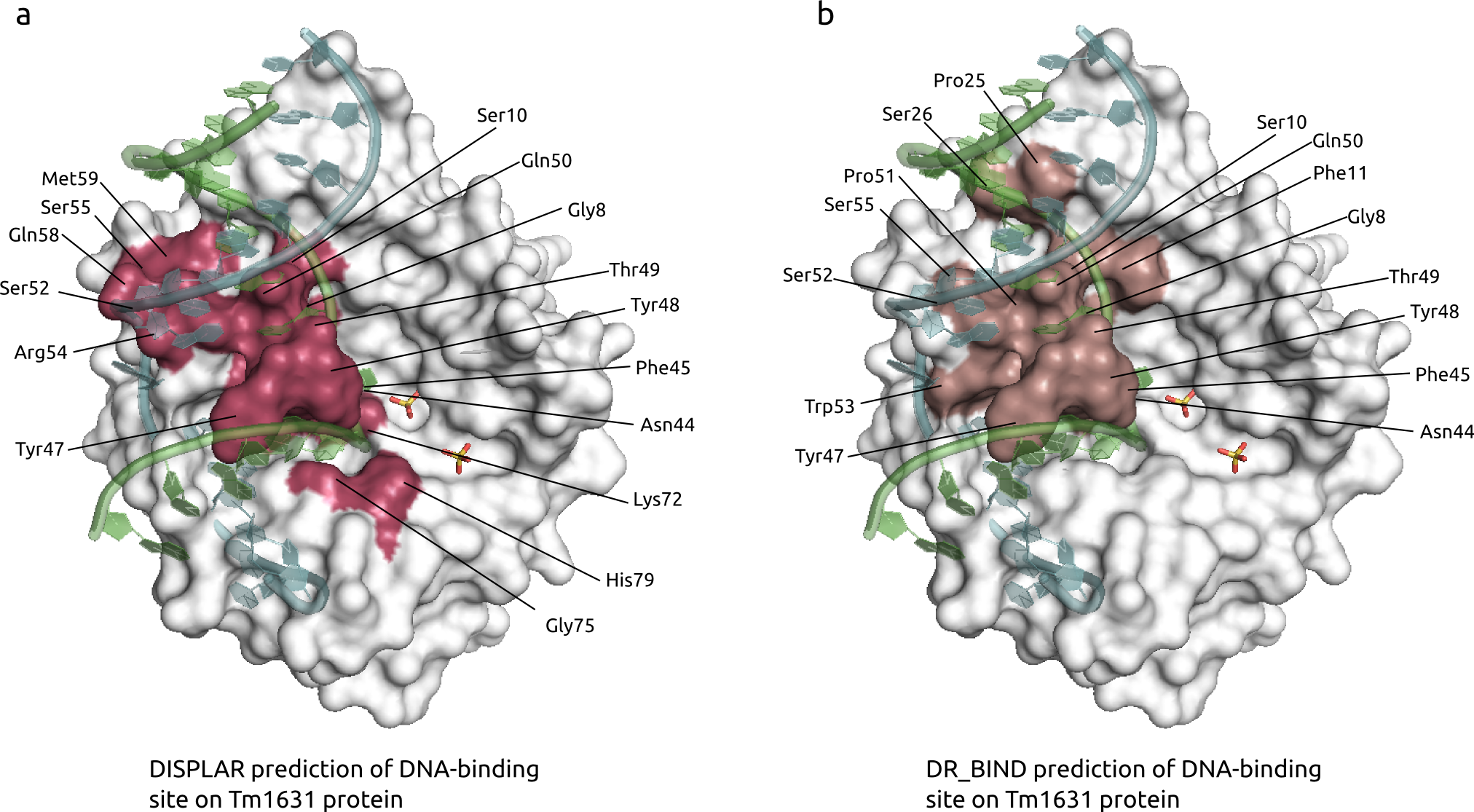


Figure S6. Prediction of DNA binding site on the Tm1631 protein. The Tm1631 is shown in surface representation (white, brown, and raspberry color). The DNA binding sites amino acid residues, predicted by DISPLAR and DR_BIND residues, are indicated by arrows. DNA, green and blue, transparent cartoons, is in the position as in our initial Tm1631-DNA model, and is shown for comparison with our model only. (a) DISPLAR prediction of DNA binding site on Tm1631 (raspberry surface). (b) DR_BIND prediction of DNA binding site on Tm1631 (brown surface).

Further, in our model, we postulated that two tyrosines are central to binding to extra-helical region of the DNA. To learn whether this two-tyrosine motif is present in any other DNA-binding proteins, we performed multiple sequence alignment using BLAST [6] of endonuclease IV (2nqj) sequence against the entire non-redundant protein sequences database. We found that tyrosines frequently occur at the same positions in endonucleases, and, specifically, in apurinic endonuclease (AP-endo1) of *Bifidobacterium*, two tyrosines appeared at the same positions as Arg37 and Tyr72 in the sequence of endonuclease IV. In Figure S7, the two tyrosines in the Tm1631 (Tyr47 and Tyr48) that bind to the extra-helical DNA region in our Tm1631-DNA model are aligned structurally to the known extra-helical DNA region-binding residues in endonuclease IV (EndoIV), Arg37 and Tyr72, (Uniprot: P0A6C1; PDB: 2nqj). These residues, in turn, are aligned with Tyr39 and Tyr72 in apurinic endonuclease (AP-endo1) (Uniprot: I3B1R5; PDB not available) according to the BLAST alignment of endonuclease IV and apurinic endonuclease sequences.


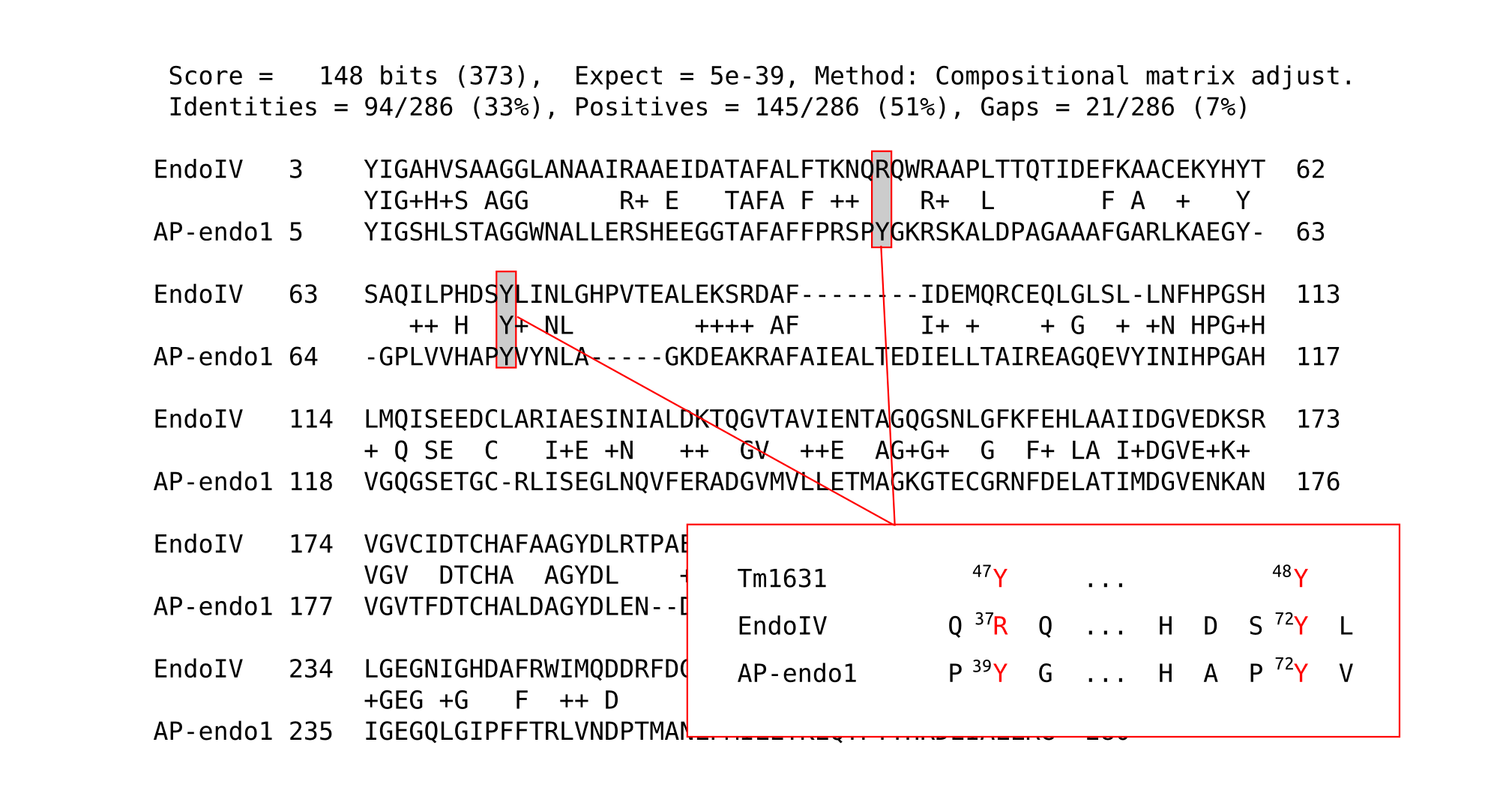


Figure S7. Sequence alignment between endonuclease IV (EndoIV) and apurinic endonuclease (AP-endo1). A new extra-helical region binding two-tyrosine motif (Tyr39 and Tyr72) in AP-endo1 is found. Aligned extra-helical region binding residues are shaded in sequences of EndoIV and AP-endo1, and their alignment (red) is shown magnified in the red box, where additionally, Tm1631 residues Tyr47 and Tyr48, are shown.

# Proposed active site in Tm1631

Proposed active site in Tm1631 bound to the reactive phosphate of the AP site in DNA is shown in Figure S8. We performed a restrained MD starting from the last frame of Tm1631-DNA model (90 ns). In this simulation, we first forced the reactive phosphate group to the predicted active site in Tm1631. Restraints were (1) between reactive phosphate group and Arg191, and (2) between reactive phosphate group and Arg145, using RESD command in CHARMM with the restraining force (kval) of 1.0 and required distance (rval) of 3.0. These restraints were applied for one nanosecond of MD. Then, the restraints were removed gradually (2nd ns kval was 0.5; 3^rd^ ns kval was 0.1), and from there we ran unrestrained MD simulation for 5 ns to equilibrate the new Tm1631-DNA model. The reactive phosphate group was firmly bound by hydrogen bonds to Lys72, Arg191, and Arg145 throughout the last 5 ns of simulation. The two glutamates, Glu143 and Glu42, bound to the arginines and seemed to stabilize the positive charges on the arginines. This could enable Arg145 and Arg191 to polarize the reactive phosphate group in DNA similar to the mechanism seen in adenylate kinases [7].


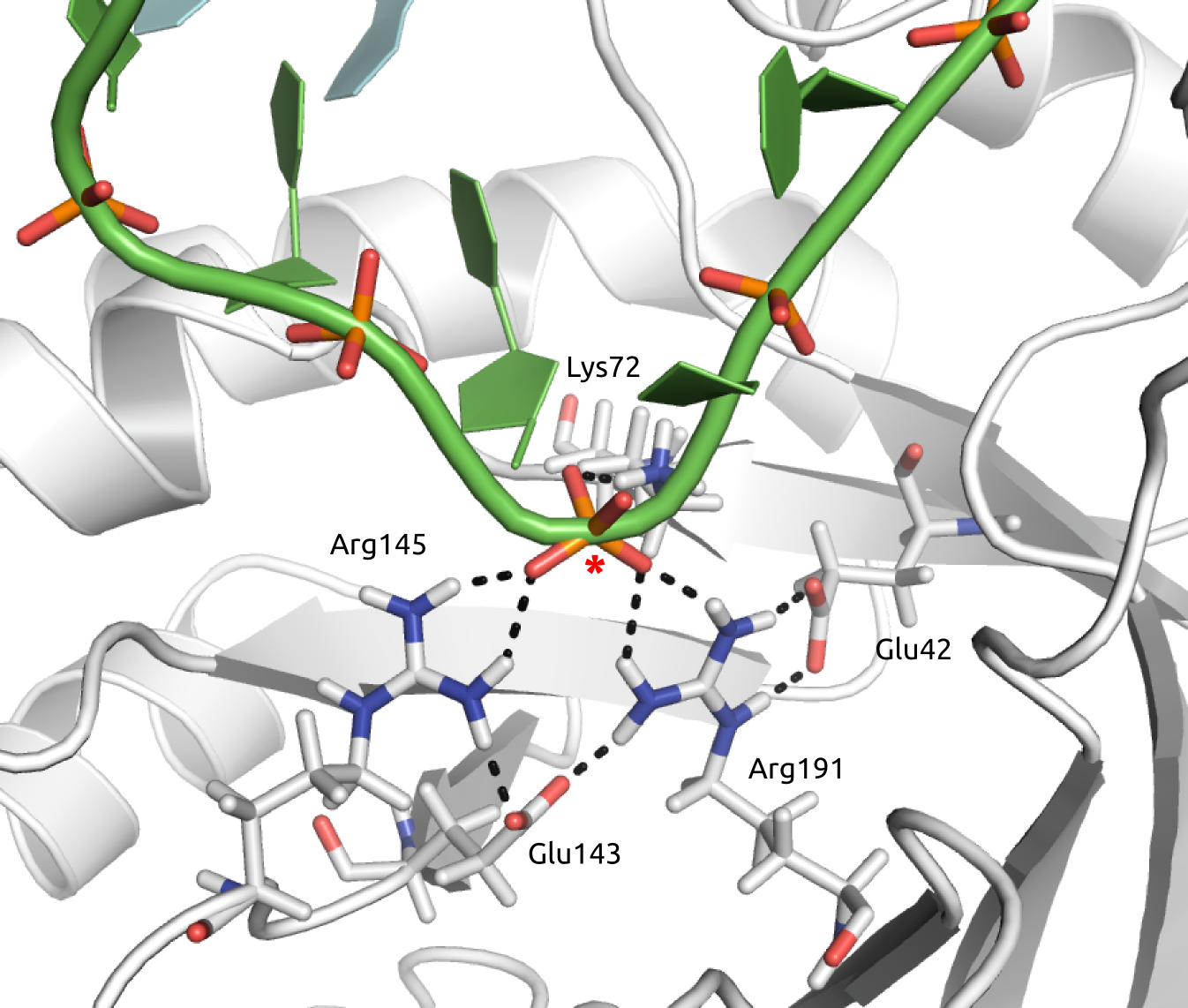


Figure S8. Proposed active site in Tm1631 with bound DNA. The reactive phosphate group is in the center and is indicated with red asterisk. Hydrogen bonds are black dashed lines.

# Binding site comparison

The similar binding sites found using ProBiS are shown in Table S1. Out of the 75 similar binding sites found with Z-score>0.5 there were 16 nucleic acids (DNA or RNA) binding sites and 15 nucleotide binding sites.

Table S1. Similar binding sites found in the non-redundant PDB using Tm1631 predicted binding site as query with ProBiS.

| PDB ID | Chain ID | Z-score |  |  |  |  | Description |  |  | Keywords |
| --- | --- | --- | --- | --- | --- | --- | --- | --- | --- | --- |
| 3qrf | H | 1,1 |  |  |  |  | Structure of a domain-swapped FOXP3 dimer |  |  | DNA BINDING PROTEIN/DNA |
| 2w9m | B | 0,98 |  |  |  |  | STRUCTURE OF FAMILY X DNA POLYMERASE FROM DEINOCOCCUS RADIODURANS |  |  | DNA REPLICATION |
| 3zte | S | 0,9 |  |  |  |  | CRYSTAL STRUCTURE OF THE TRP RNA-BINDING ATTENUATION PROTEIN (TRAP) FROM BACILLUS LICHENIFORMIS. |  |  | RNA-BINDING PROTEIN |
| 2jjx | B | 0,9 |  |  |  |  | THE CRYSTAL STRUCTURE OF UMP KINASE FROM BACILLUS ANTHRACIS (BA1797) |  |  | TRANSFERASE |
| 2vy0 | A | 0,89 |  |  |  |  | THE X-RAY STRUCTURE OF ENDO-BETA-1,3-GLUCANASE FROM PYROCOCCUS FURIOSUS |  |  | HYDROLASE |
| 3fhf | A | 0,88 |  |  |  |  | Crystal structure of Methanocaldococcus jannaschii 8-oxoguanine DNA glycosylase (MjOgg) |  |  | DNA repair, HYDROLASE, LYASE |
| 1nsc | B | 0,87 |  |  |  |  | INFLUENZA B VIRUS NEURAMINIDASE CAN SYNTHESIZE ITS OWN INHIBITOR |  |  | HYDROLASE(O-GLYCOSYL) |
| 1lvg | A | 0,81 |  |  |  |  | Crystal structure of mouse guanylate kinase in complex with GMP and ADP |  |  | TRANSFERASE |
| 1nio | A | 0,8 |  |  |  |  | Crystal structure of beta-luffin, a ribosome inactivating protein at 2.0A resolution |  |  | hydrolase |
| 3zzs | C | 0,79 |  |  |  |  | ENGINEERED 12-SUBUNIT BACILLUS STEAROTHERMOPHILUS TRP RNA-BINDING ATTENUATION PROTEIN (TRAP) |  |  | TRANSCRIPTION |
| 3ecc | A | 0,79 |  |  |  |  | Crystal structure of the DnaC helicase loader in complex with ADP-BeF3 |  |  | REPLICATION |
| 3ju0 | A | 0,75 |  |  |  |  | Structure of the arm-type binding domain of HAI7 integrase |  |  | DNA BINDING PROTEIN |
| 3ilz | A | 0,73 |  |  |  |  | Structure of TR-alfa bound to selective thyromimetic GC-1 in P212121 space group |  |  | SIGNALING PROTEIN |
| 3iln | A | 0,72 |  |  |  |  | X-ray structure of the laminarinase from Rhodothermus marinus |  |  | HYDROLASE |
| 3fiq | B | 0,72 |  |  |  |  | Odorant Binding Protein OBP1 |  |  | TRANSPORT PROTEIN |
| 3h8k | A | 0,71 |  |  |  |  | Crystal structure of Ube2g2 complxed with the G2BR domain of gp78 at 1.8-A resolution |  |  | LIGASE |
| 3dj6 | A | 0,71 |  |  |  |  | Crystal structure of the mouse Aurora-A catalytic domain (Asn186-&#62 |  |  | Leu) in complex with Compound 823. |
| 3bso | A | 0,71 |  |  |  |  | Norwalk Virus polymerase bound to cytidine 5'-triphosphate and primer-template RNA |  |  | Transferase/RNA |
| 2q3o | A | 0,71 |  |  |  |  | Ensemble refinement of the protein crystal structure of 12-oxo-phytodienoate reductase isoform 3 |  |  | OXIDOREDUCTASE |
| 1b04 | A | 0,71 |  |  |  |  | STRUCTURE OF THE ADENYLATION DOMAIN OF AN NAD+ DEPENDENT LIGASE |  |  | LIGASE |
| 3dgt | A | 0,7 |  |  |  |  | The 1.5 A crystal structure of endo-1,3-beta-glucanase from Streptomyces sioyaensis |  |  | HYDROLASE |
| 1nko | A | 0,7 |  |  |  |  | Energetic and structural basis of sialylated oligosaccharide recognition by the natural killer cell inhibitory receptor p75/AIRM1 or Siglec-7 |  |  | IMMUNE SYSTEM |
| 3atg | A | 0,69 |  |  |  |  | endo-1,3-beta-glucanase from Cellulosimicrobium cellulans |  |  | HYDROLASE |
| 2qtr | C | 0,69 |  |  |  |  | Crystal Structure of Nicotinate Mononucleotide Adenylyltransferase |  |  | TRANSFERASE |
| 1yro | C | 0,69 |  |  |  |  | Crystal structure of beta14,-galactosyltransferase mutant ARG228Lys in complex with alpha-lactalbumin in the presence of UDP-galactose and Mn |  |  | TRANSFERASE ACTIVATOR/TRANSFERASE |
| 3j21 | f | 0,68 |  |  |  |  | Promiscuous behavior of proteins in archaeal ribosomes revealed by cryo-EM: implications for evolution of eukaryotic ribosomes (50S ribosomal proteins) |  |  | RIBOSOME |
| 3ogk | H | 0,67 |  |  |  |  | Structure of COI1-ASK1 in complex with coronatine and an incomplete JAZ1 degron |  |  | PROTEIN BINDING |
| 2car | A | 0,67 |  |  |  |  | CRYSTAL STRUCTURE OF HUMAN INOSINE TRIPHOSPHATASE |  |  | HYDROLASE |
| 3q2o | A | 0,66 |  |  |  |  | Crystal Structure of purK: N5-carboxyaminoimidazole ribonucleotide synthetase |  |  | LYASE |
| 3ieh | A | 0,66 |  |  |  |  | Crystal structure of Putative metallopeptidase (YP_001051774.1) from SHEWANELLA BALTICA OS155 at 2.45 A resolution |  |  | HYDROLASE |
| 1am5 | A | 0,66 |  |  |  |  | THE CRYSTAL STRUCTURE AND PROPOSED AMINO ACID SEQUENCE OF A PEPSIN FROM ATLANTIC COD (GADUS MORHUA) |  |  | ASPARTYL PROTEASE |
| 4e4t | B | 0,65 |  |  |  |  | Crystal structure of Phosphoribosylaminoimidazole carboxylase, ATPase subunit from Burkholderia ambifaria |  |  | LYASE |
| 3q2k | G | 0,65 |  |  |  |  | Crystal structure of the WlbA dehydrogenase from Bordetella pertussis in complex with NADH and UDP-GlcNAcA |  |  | OXIDOREDUCTASE |
| 3azg | G | 0,65 |  |  |  |  | Crystal Structure of Human Nucleosome Core Particle Containing H3K115Q mutation |  |  | STRUCTURAL PROTEIN/DNA |
| 4ibo | C | 0,64 |  |  |  |  | Crystal structure of a putative gluconate dehydrogenase from agrobacterium tumefaciens (target EFI-506446) |  |  | OXIDOREDUCTASE |
| 2r4v | A | 0,64 |  |  |  |  | Structure of human CLIC2, crystal form A |  |  | TRANSPORT PROTEIN |
| 2ihm | B | 0,64 |  |  |  |  | Polymerase mu in ternary complex with gapped 11mer DNA duplex and bound incoming nucleotide |  |  | Transferase/DNA |
| 2a07 | I | 0,64 |  |  |  |  | Crystal Structure of Foxp2 bound Specifically to DNA. |  |  | Transcription/DNA |
| 1zd1 | A | 0,64 |  |  |  |  | Human Sulfortransferase SULT4A1 |  |  | TRANSFERASE |
| 2hei | B | 0,63 |  |  |  |  | Crystal structure of human RAB5B in complex with GDP |  |  | TRANSPORT PROTEIN |
| 1zdn | B | 0,63 |  |  |  |  | Ubiquitin-conjugating enzyme E2S |  |  | Ligase |
| 3r8t | J | 0,62 |  |  |  |  | Structures of the bacterial ribosome in classical and hybrid states of tRNA binding |  |  | RIBOSOME |
| 3his | A | 0,62 |  |  |  |  | Crystal structure of Saporin-L1 from Saponaria officinalis |  |  | HYDROLASE |
| 2jep | B | 0,62 |  |  |  |  | NATIVE FAMILY 5 XYLOGLUCANASE FROM PAENIBACILLUS PABULI |  |  | HYDROLASE |
| 2a0a | A | 0,62 |  |  |  |  | Solution Structure of Der f 13, Group 13 Allergen from House Dust Mites |  |  | ALLERGEN |
| 1pjc | A | 0,62 |  |  |  |  | L-ALANINE DEHYDROGENASE COMPLEXED WITH NAD |  |  | OXIDOREDUCTASE |
| 1f82 | A | 0,62 |  |  |  |  | BOTULINUM NEUROTOXIN TYPE B CATALYTIC DOMAIN |  |  | TOXIN,HYDROLASE |
| 3q72 | B | 0,61 |  |  |  |  | Crystal Structure of Rad G-domain-GTP Analog Complex |  |  | SIGNALING PROTEIN |
| 3bfx | A | 0,61 |  |  |  |  | Crystal structure of human sulfotransferase SULT1C1 in complex with PAP |  |  | TRANSFERASE |
| 2bfd | A | 0,61 |  |  |  |  | REACTIVITY MODULATION OF HUMAN BRANCHED-CHAIN ALPHA-KETOACID DEHYDROGENASE BY AN INTERNAL MOLECULAR SWITCH |  |  | OXIDOREDUCTASE |
| 1t3f | B | 0,61 |  |  |  |  | THREE DIMENSIONAL STRUCTURE OF A HUMANIZED ANTI-IFN-GAMMA FAB (HuZAF) IN P21 21 21 SPACE GROUP |  |  | IMMUNE SYSTEM |
| 1ogy | J | 0,61 |  |  |  |  | CRYSTAL STRUCTURE OF THE HETERODIMERIC NITRATE REDUCTASE FROM RHODOBACTER SPHAEROIDES |  |  | OXIDOREDUCTASE |
| 1e8c | A | 0,61 |  |  |  |  | STRUCTURE OF MURE THE UDP-N-ACETYLMURAMYL TRIPEPTIDE SYNTHETASE FROM E. COLI |  |  | LIGASE |
| 4g0a | D | 0,6 |  |  |  |  | Crystallographic Analysis of Rotavirus NSP2-RNA Complex Reveals Specific Recognition of 5'-GG Sequence for RTPase activity |  |  | HYDROLASE/RNA |
| 2zgm | B | 0,6 |  |  |  |  | Crystal structure of recombinant Agrocybe aegerita lectin,rAAL, complex with lactose |  |  | Hydrolase |
| 1pby | A | 0,59 |  |  |  |  | Structure of the Phenylhydrazine Adduct of the Quinohemoprotein Amine Dehydrogenase from Paracoccus denitrificans at 1.7 A Resolution |  |  | OXIDOREDUCTASE |
| 1ls9 | A | 0,59 |  |  |  |  | Structure of the Cytochrome c6 from the Green Alga Cladophora glomerata |  |  | ELECTRON TRANSPORT |
| 2hyk | A | 0,58 |  |  |  |  | The crystal structure of an endo-beta-1,3-glucanase from alkaliphilic Nocardiopsis sp.strain F96 |  |  | HYDROLASE |
| 3u5g | R | 0,57 |  |  |  |  | The structure of the eukaryotic ribosome at 3.0 A resolution. This entry contains proteins of the 40S subunit, ribosome B |  |  | RIBOSOME |
| 3r4z | A | 0,57 |  |  |  |  | Crystal structure of alpha-neoagarobiose hydrolase (ALPHA-NABH) in complex with alpha-d-galactopyranose from Saccharophagus degradans 2-40 |  |  | HYDROLASE |
| 2q4z | A | 0,57 |  |  |  |  | Ensemble refinement of the protein crystal structure of an aspartoacylase from Rattus norvegicus |  |  | HYDROLASE |
| 3syj | A | 0,56 |  |  |  |  | Crystal structure of the Haemophilus influenzae Hap adhesin |  |  | CELL ADHESION |
| 2poz | F | 0,56 |  |  |  |  | Crystal structure of a putative dehydratase from Mesorhizobium loti |  |  | STRUCTURAL GENOMICS, UNKNOWN FUNCTION |
| 1g5t | A | 0,56 |  |  |  |  | THE THREE-DIMENSIONAL STRUCTURE OF ATP:CORRINOID ADENOSYLTRANSFERASE FROM SALMONELLA TYPHIMURIUM. APO-ATP FORM |  |  | TRANSFERASE |
| 3uxy | A | 0,55 |  |  |  |  | The crystal structure of short chain dehydrogenase from Rhodobacter sphaeroides |  |  | OXIDOREDUCTASE |
| 3s2j | A | 0,55 |  |  |  |  | Crystal structure of dipeptidase from Streptomyces coelicolor complexed with phosphinate pseudodipeptide L-Leu-D-Ala |  |  | HYDROLASE/HYDROLASE INHIBITOR |
| 3vg9 | A | 0,54 |  |  |  |  | Crystal structure of human adenosine A2A receptor with an allosteric inverse-agonist antibody at 2.7 A resolution |  |  | SIGNALING PROTEIN |
| 3k80 | B | 0,54 |  |  |  |  | Structure of essential protein from Trypanosoma brucei |  |  | Immune System, RNA Binding Protein |
| 2wpg | A | 0,53 |  |  |  |  | Sucrose Hydrolase |  |  | HYDROLASE |
| 2wl9 | A | 0,52 |  |  |  |  | CRYSTAL STRUCTURE OF CATECHOL 2,3-DIOXYGENASE |  |  | OXIDOREDUCTASE |
| 2gl5 | A | 0,52 |  |  |  |  | Crystal Structure of Putative Dehydratase from Salmonella Thyphimurium |  |  | STRUCTURAL GENOMICS, UNKNOWN FUNCTION |
| 3k8t | A | 0,51 |  |  |  |  | Structure of eukaryotic rnr large subunit R1 complexed with designed adp analog compound |  |  | OXIDOREDUCTASE |
| 2bpd | A | 0,51 |  |  |  |  | STRUCTURE OF MURINE DECTIN-1 |  |  | RECEPTOR |
| 1qw9 | A | 0,51 |  |  |  |  | Crystal structure of a family 51 alpha-L-arabinofuranosidase in complex with 4-nitrophenyl-Ara |  |  | HYDROLASE |

# Validation of binding site comparison as function prediction approach

1. We validated our function prediction approach on 369 well-characterized proteins from the ligAsite benchmark set of biologically relevant binding sites in proteins of known apo- and holo-structures [4]. To simulate the conditions of the function prediction of unknown Tm1631 protein, where no homologous structures were available in the PDB, we excluded all such homologous proteins from the comparison set, that is, the non-redundant PDB (nr-PDB) of representatives >95% sequence identical protein structures. Specifically, for each benchmark protein we created a unique comparison set, by including to it only protein structures that have very little sequence identity (bit score of <32) with the corresponding benchmark, where sequence identity was calculated using BLAST sequence alignment tool [5]. We chose the threshold of 32 bits, since this is the BLAST score of Tm1631 protein's (PDB: 1vpq) most similar protein sequence with known function in the nr-PDB. Thereby, we excluded from each benchmark protein's comparison set all proteins with their sequences similar to the sequence of Tm1631 protein. Function prediction under such conditions is hard, since proteins from which function could easily be transferred based on sequence alignment are excluded.
2. To predict the functions of the benchmark proteins in their apo forms, i.e., without the bound ligand, we followed the function prediction approach (see Methods) used for unknown Tm1631 protein with the difference that for each benchmark protein we considered only its PDB structure, i.e., one frame. We defined the binding site on the benchmark protein using the binding site residue numbers found in the ligAsite database. Next, we compared each benchmark protein's binding site as defined by the ligAsite database with the corresponding comparison set of protein structures using ProBiS algorithm. We used LOCAL and MOTIF options, to restrict comparison to the binding site only and to define the binding site using residue numbers, respectively. These comparisons resulted in lists of locally similar nr-PDB proteins, one such list for each benchmark protein. Each similar protein in a list had a surface patch in common with the benchmark protein's binding site. We then determined if the similar surface patches found belong to a binding site in the similar protein, by transposing to the similar protein all ligands found in its >30% sequence identical proteins from the entire PDB. The binding site was then defined as residues <3 Å around the cluster of ligands. We ranked the similar proteins resulted from the above described procedure using their Z-scores and considered the function of the highest scoring similar protein as function prediction of the benchmark protein.
3. To see if the predicted function and the known function of the benchmark protein agree, i.e., if the function was correctly predicted, we then determined the function identifiers for each pair of the benchmark protein and its corresponding highest scoring similar protein, i.e., Pfam number, EC number, GO terms, using the SIFTS database [6]. Alternatively, if one of the proteins did not have any function identifiers, and if ligands were available in both proteins, we downloaded their respective ligand structures as PDB files from the PDB web site. We compared the function identifiers of the benchmark protein and the highest scoring similar protein as strings using a simple text search in the following order: Pfam, EC number, and GO terms; if we found a match between at least one pair of the identifiers, we considered that the function of the benchmark protein was correctly predicted. We compared ligands using an in-house developed algorithm based on graph representation of molecules. The algorithm compared a pair of ligands and returned their level of similarity expressed as the Tanimoto coefficient (T). We considered that the function of the benchmark protein was correctly predicted if T>0.6, where T ranges between 0 (ligands share no similar substructure) and 1 (identical ligands).

Table S2. Validation of binding site comparison for function prediction on a set of 369 benchmark PDB protein structures with known functions using ProBiS algorithm. Asterisk (*) in the first column indicates that function of the benchmark protein was correctly predicted, i.e., the benchmark and the similar protein have at least one function identifier (see Pfam, EC, GO, and Ligand columns) in common.

| Funct.  Match | Benchmark PDB.CHAIN | Similar PDB.CHAIN | Pfam number | EC number | GO term | Ligand  #1.#2.Tanimoto | ProBiS Z-score | BLAST  bit-score | BLAST expect. |
| --- | --- | --- | --- | --- | --- | --- | --- | --- | --- |
| * | 180l.A | 1q7l.A |  |  | 8152 |  | 1.62 | 18 | 0.083 |
| * | 1a4u.A | 3ak4.A |  |  | 8152 | NAD.NAD.1 | 2.62 | 32 | 6e-06 |
|  | 1ade.A | 2i0i.C |  |  |  |  | 1.27 | 16 | 0.29 |
|  | 1ak1.A | 2voh.B |  |  |  |  | 1.3 |  |  |
| * | 1akz.A | 2owr.D | PF03167 |  | 6281 |  | 1.6 | 29 | 3e-05 |
| * | 1arb.A | 2sfa.A |  |  | 3824 |  | 2.26 | 19 | 0.072 |
| * | 1arl.A | 4axv.A |  |  | 4181 | ZN.ZN.1 | 2.21 | 17 | 0.42 |
| * | 1az3.A | 2oaa.A |  |  | 46872 |  | 1.46 | 13 | 4.7 |
| * | 1az5.A | 3liy.D | PF00077 |  | 4190 |  | 1.97 | 28 | 4e-06 |
|  | 1b8e.A | 3i84.B |  |  |  |  | 1.57 |  |  |
| * | 1b8p.A | 2v65.A | PF00056 |  | 3824 |  | 2.42 | 28 | 2e-04 |
| * | 1bd9.A | 2evv.A | PF01161 |  |  |  | 1.95 | 25 | 4e-04 |
|  | 1bec.A | 3hvu.C |  |  |  |  | 0.72 | 15 | 1.4 |
| * | 1bk7.A | 4dw5.B |  |  | 3723 |  | 1.66 | 28 | 3e-05 |
| * | 1bkz.A | 2wkk.C | PF00337 |  | 5576 |  | 2.52 | 30 | 2e-06 |
| * | 1bqc.A | 1pbg.B |  |  | 3824 | BMA.BGP.0.75 | 1.4 | 18 | 0.55 |
| * | 1byi.A | 3fgn.C |  | 6.3.3.3 | 166 | DAA.DSD.0.8 | 2.36 | 23 | 0.003 |
|  | 1c48.B | 2iw3.A |  |  |  |  | 1.28 | 17 | 0.27 |
|  | 1c5h.A | 4b29.A |  |  |  |  | 1.04 | 17 | 0.34 |
| * | 1ceo.A | 2whl.A | PF00150 |  | 3824 | BGC.MAN.1 | 1.87 | 13 | 8.9 |
| * | 1cex.A | 2czq.A | PF01083 |  | 8152 |  | 2.3 | 28 | 6e-05 |
| * | 1cpj.A | 1zcm.A |  |  | 6508 |  | 2.23 | 23 | 0.008 |
| * | 1crw.G | 2g76.A |  |  | 51287 | NAD.NAD.1 | 2.12 | 25 | 0.002 |
| * | 1cwy.A | 3dc0.A |  |  | 3824 |  | 1.99 | 20 | 0.21 |
| * | 1dco.A | 3jtj.A |  |  | 5737 |  | 1.21 |  |  |
|  | 1dhn.A | 3mqz.A |  |  |  |  | 1.71 | 15 | 0.56 |
| * | 1dq0.A | 2gud.B |  |  | 5537 | AMG.BGC.0.923077 | 1.38 | 13 | 2.7 |
| * | 1dup.A | 2bsy.A | PF00692 | 3.6.1.23 | 4170 |  | 1.5 | 25 | 3e-04 |
| * | 1e4f.T | 4ehu.B |  |  |  | ATP.ADP.0.870968 | 1.83 | 18 | 0.31 |
| * | 1e5l.A | 1dpg.B |  |  | 16491 | NDP.NAD.0.916667 | 1.86 | 18 | 0.7 |
| * | 1e8y.A | 1cja.B |  |  | 16772 |  | 2.06 | 17 | 2.7 |
| * | 1edq.A | 3fnd.A | PF00704 |  | 3824 |  | 2.2 | 22 | 0.029 |
| * | 1epa.A | 1gm6.A | PF00061 |  | 5215 |  | 1.39 | 26 | 1e-04 |
| * | 1ewz.A | 2cc1.A |  | 3.5.2.6 | 8800 |  | 1.58 | 17 | 0.34 |
|  | 1ey0.A | 1nhs.A |  |  |  |  | 1.88 | 16 | 0.82 |
|  | 1f14.A | 2hmt.A |  |  |  |  | 2.09 | 25 | 7e-04 |
|  | 1f1s.A | 3let.B |  |  |  |  | 1.08 | 18 | 1 |
|  | 1f2v.A | 1o7e.B |  |  |  |  | 1.4 |  |  |
|  | 1f41.A | 2qkd.A |  |  |  |  | 1.87 |  |  |
|  | 1f5z.A | 4dnh.A |  |  |  |  | 1.37 | 15 | 3.5 |
|  | 1fcq.A | 3nn8.G |  |  |  |  | 1.43 | 19 | 0.04 |
| * | 1fgb.D | 1y9l.A |  |  | 9405 |  | 1.59 | 25 | 7e-05 |
|  | 1fo9.A | 3b6e.A |  |  |  |  | 1.6 |  |  |
| * | 1fsf.A | 1y89.B | PF01182 |  | 5975 |  | 1.69 | 32 | 8e-06 |
| * | 1ftf.B | 2wds.A | PF01648 | 2.7.8.7 | 287 |  | 1.64 | 27 | 3e-05 |
|  | 1ftr.A | 2zd7.B |  |  |  |  | 1.83 | 14 | 3.3 |
| * | 1fwl.A | 4ed4.A | PF00288 |  | 166 | ADP.ATP.0.870968 | 1.67 |  |  |
|  | 1g40.A | 2h9e.C |  |  |  |  | 1.73 | 14 | 0.83 |
| * | 1g4e.B | 3ceu.A | PF02581 |  | 3824 |  | 1.35 | 23 | 0.003 |
| * | 1g95.A | 3brk.X | PF00483 |  | 9058 |  | 2.14 | 27 | 0.001 |
| * | 1gbs.A | 1qsa.A | PF01464 |  |  | BUL.BLG.1 | 2.05 | 22 | 0.012 |
| * | 1gce.A | 1e25.A | PF00144 |  | 8800 |  | 1.55 | 14 | 4.1 |
| * | 1gfs.A | 1wvg.B |  |  | 3824 | NAP.APR.0.714286 | 2.07 | 24 | 0.003 |
| * | 1gou.A | 1lni.B | PF00545 |  | 3723 |  | 2.67 | 28 | 7e-06 |
| * | 1gsh.A | 3df7.A |  |  | 5524 |  | 2.3 | 18 | 0.27 |
|  | 1gwk.A | 3gzx.B |  |  |  |  | 1.29 | 13 | 4.1 |
| * | 1gy0.A | 3u0j.A | PF01129 |  |  |  | 2.25 | 23 | 0.004 |
|  | 1hk9.A | 2qtx.J |  |  |  |  | 1.85 | 25 | 2e-05 |
|  | 1hka.A | 1vr4.A |  |  |  |  | 1.69 |  |  |
|  | 1ho1.A | 3rcm.A |  |  |  |  | 1.33 | 15 | 2.4 |
|  | 1i7n.A | 1ysj.A |  |  |  |  | 1.11 | 14 | 5.8 |
| * | 1iad.A | 3k7n.A |  |  | 4222 | ZN.ZN.1 | 2.24 | 23 | 0.004 |
|  | 1ilv.A | 3r6d.A |  |  |  |  | 1.51 |  |  |
| * | 1inl.A | 3c6k.D | PF01564 |  | 3824 | AAT.MTA.0.689655 | 2.21 | 30 | 5e-05 |
| * | 1j85.A | 3ic6.A | PF00588 |  | 3723 |  | 2.07 | 24 | 9e-04 |
|  | 1j8s.A | 3qum.H |  |  |  |  | 1.14 | 15 | 1.4 |
|  | 1jcf.A | 3mdq.A |  |  |  |  | 1.93 | 23 | 0.011 |
| * | 1jks.A | 1csn.A | PF00069 |  | 4672 | ADP.ATP.0.870968 | 2.85 | 32 | 6e-06 |
|  | 1jxo.A | 1z3e.B |  |  |  |  | 1.09 |  |  |
| * | 1jyk.A | 1hm9.A | PF00483 |  | 9058 | CDC.UD1.0.666667 | 1.82 | 28 | 3e-04 |
|  | 1k0m.A | 4g10.A |  |  |  |  | 1.71 | 28 | 7e-05 |
|  | 1k3o.A | 3bpq.A |  |  |  |  | 1.62 |  |  |
| * | 1k6a.A | 3vdh.B |  |  | 3824 |  | 1.59 | 17 | 0.58 |
| * | 1kam.A | 1vlh.A | PF01467 |  | 166 |  | 2.21 | 19 | 0.028 |
| * | 1kf5.A | 3snf.A | PF00074 |  | 3676 | 5GP.AMP.0.88 | 2.37 | 29 | 3e-06 |
|  | 1khd.B | 3rfb.A |  |  |  |  | 0.86 | 17 | 0.28 |
|  | 1kn9.A | 3a5z.F |  |  |  |  | 1.23 | 13 | 3.9 |
| * | 1kpa.A | 1fit.A | PF01230 |  | 166 |  | 2.2 | 32 | 5e-07 |
| * | 1kwb.B | 3lm4.B | PF00903 |  | 16491 |  | 1.98 | 26 | 8e-04 |
| * | 1l7d.A | 2v65.A |  |  | 16491 |  | 1.95 | 18 | 0.4 |
| * | 1lbv.A | 2q74.A | PF00459 | 3.1.3.25 | 8934 |  | 2.28 | 32 | 6e-06 |
|  | 1lci.A | 2fl7.A |  |  |  |  | 1.01 | 15 | 2.7 |
| * | 1lf4.A | 3kt2.A |  |  | 4190 |  | 1.49 | 14 | 3.1 |
|  | 1ltu.A | 2xpp.B |  |  |  |  | 1.17 | 15 | 0.14 |
| * | 1m1z.A | 2hma.A |  |  | 166 | MG.MG.1 | 2 | 20 | 0.19 |
| * | 1mkb.A | 3u5c.V |  |  | 5737 |  | 1.02 |  |  |
|  | 1mmi.A | 2x3g.A |  |  |  |  | 1.35 | 13 | 2.7 |
| * | 1mr7.A | 3fsy.E |  |  | 16740 | ACO.SCA.0.927273 | 1.63 | 14 | 3.2 |
|  | 1mtz.A | 1fwx.D |  |  |  |  | 0.56 | 15 | 4.3 |
| * | 1mwk.A | 3kvg.A |  |  |  | ADP.ADP.1 | 1.51 |  |  |
|  | 1mzl.A | 1z8y.O |  |  |  |  | 1.71 |  |  |
| * | 1n05.A | 1txl.A |  |  |  | ZN.ZN.1 | 1.82 | 16 | 0.48 |
|  | 1ndb.A | 3tva.B |  |  |  |  | 1.74 | 18 | 0.38 |
|  | 1noa.A | 2pbc.C |  |  |  |  | 1.88 |  |  |
| * | 1non.A | 1vdm.B | PF00156 |  | 9116 |  | 2.29 | 30 | 8e-06 |
|  | 1nxm.A | 2wel.A |  |  |  |  | 1.67 | 20 | 0.055 |
|  | 1o24.A | 3hie.B |  |  |  |  | 1.73 | 14 | 1.5 |
| * | 1oem.X | 1rxd.B | PF00102 |  | 4725 |  | 1.56 | 29 | 2e-05 |
|  | 1ofp.A | 3gmf.A |  |  |  |  | 0.75 |  |  |
| * | 1ogh.A | 3lqw.A | PF00692 |  | 16787 |  | 2.15 | 25 | 4e-04 |
|  | 1ogl.A | 1q3o.A |  |  |  |  | 1.62 |  |  |
| * | 1ogm.X | 1hg8.A |  |  | 5576 |  | 1.21 | 18 | 0.68 |
| * | 1ojq.A | 2gwm.A |  |  | 5576 | NAD.NAD.1 | 1.87 | 14 | 1.6 |
|  | 1ooi.X | 1ofu.X |  |  |  |  | 1.32 |  |  |
| * | 1opy.A | 3rga.A |  |  | 16853 |  | 1.88 | 29 | 1e-05 |
| * | 1oxt.A | 3qf7.A |  |  | 166 | ADP.ADP.1 | 2.69 | 32 | 2e-05 |
| * | 1p1x.A | 1kbi.B |  |  | 3824 |  | 1.32 | 16 | 2.3 |
| * | 1p5h.A | 2yy8.B |  |  | 5737 |  | 0.86 | 20 | 0.059 |
|  | 1p6z.R | 2atm.A |  |  |  |  | 2.15 | 13 | 2.5 |
| * | 1p74.A | 1gpj.A | PF01488 |  | 16491 |  | 1.32 | 32 | 1e-05 |
| * | 1pdb.A | 2p4g.A |  |  | 55114 |  | 2.16 | 22 | 0.01 |
| * | 1png.A | 3ks7.A |  |  | 3824 |  | 1.69 | 32 | 1e-05 |
|  | 1q52.A | 3gwk.E |  |  |  |  | 1.16 | 18 | 0.057 |
| * | 1q7m.A | 3mcm.A | PF00809 |  | 42558 |  | 2.06 | 20 | 0.24 |
|  | 1qcz.A | 2vjh.C |  |  |  |  | 1.25 | 19 | 0.033 |
| * | 1qid.A | 2o7r.A |  |  | 4091 |  | 1.88 | 29 | 2e-04 |
|  | 1qto.A | 2ogx.B |  |  |  |  | 1.24 | 13 | 3.3 |
| * | 1qtr.A | 3o4h.A |  |  | 5737 |  | 1.97 | 18 | 0.53 |
| * | 1r0m.A | 3gc2.A | PF01188 |  | 3824 |  | 1.86 | 30 | 5e-05 |
|  | 1r12.A | 3lx1.A |  |  |  |  | 1.2 | 19 | 0.066 |
|  | 1r29.A | 4f4c.A |  |  |  |  | 1.39 | 20 | 0.094 |
|  | 1rgp.A | 3nvq.E |  |  |  |  | 0.84 | 19 | 0.24 |
| * | 1ri5.A | 2vdw.A | PF03291 |  |  | SA8.SAH.0.892857 | 2.71 | 23 | 0.008 |
| * | 1riq.A | 1x54.A |  |  | 166 | ATP.4AD.0.631579 | 2.34 | 17 | 1.1 |
|  | 1rj1.A | 1h75.A |  |  |  |  | 1.68 | 23 | 3e-04 |
|  | 1rkm.A | 1yii.A |  |  |  |  | 1.29 |  |  |
| * | 1rzv.A | 2x6q.A | PF00534 |  | 9058 | ADP.UDP.0.677419 | 1.29 | 24 | 0.008 |
| * | 1s2l.A | 2f62.B | PF05014 |  | 9159 |  | 2.16 | 22 | 0.004 |
|  | 1s7k.A | 3u5e.L |  |  |  |  | 1.87 |  |  |
| * | 1sgk.A | 1ikp.A |  | 2.4.2.36 | 16740 | NAD.APR.0.777778 | 2.45 | 32 | 6e-05 |
| * | 1sgz.A | 3kt2.A |  |  | 4190 |  | 1.65 | 15 | 1.6 |
|  | 1sjy.A | 3p8d.B |  |  |  |  | 2.28 | 19 | 0.005 |
| * | 1snt.A | 1e8u.A |  |  | 4308 | DAN.DAN.1 | 2.16 | 15 | 5.3 |
| * | 1sqg.A | 3hp7.A |  |  | 3723 |  | 2.11 | 15 | 2.7 |
| * | 1sul.A | 1jwy.B |  |  | 5525 | MG.MG.1 | 1.77 | 27 | 2e-04 |
| * | 1swh.B | 2y32.D | PF01382 |  |  |  | 1.88 | 28 | 1e-05 |
|  | 1tgn.A | 1yp8.A |  |  |  |  | 0.92 | 13 | 0.3 |
|  | 1thv.A | 1hxm.G |  |  |  |  | 0.81 | 13 | 3.3 |
| * | 1tib.A | 2qub.A | PF01764 | 3.1.1.3 | 4806 |  | 1.85 | 25 | 0.003 |
|  | 1tje.A | 3h5d.B |  |  |  |  | 1.18 | 15 | 1.5 |
|  | 1tjv.A | 3q0h.A |  |  |  |  | 1.16 |  |  |
| * | 1tvn.A | 1h1n.A | PF00150 |  | 3824 |  | 2.03 | 21 | 0.026 |
|  | 1tw0.A | 1yox.F |  |  |  |  | 1.24 | 14 | 2.2 |
|  | 1tyf.A | 3zwl.F |  |  |  |  | 1.62 | 17 | 0.017 |
|  | 1tyv.A | 1no1.B |  |  |  |  | 1.54 | 20 | 0.038 |
|  | 1tzv.A | 1xm8.B |  |  |  |  | 1.18 | 14 | 2 |
| * | 1u09.A | 2xwh.A |  |  | 3723 |  | 2.43 | 23 | 0.021 |
|  | 1u0t.A | 2bbr.A |  |  |  |  | 1.18 |  |  |
| * | 1u7u.A | 1p9o.B | PF04127 |  | 4632 |  | 2.02 | 30 | 3e-05 |
| * | 1uaj.A | 1ryp.A |  |  | 5737 |  | 1.21 | 17 | 0.44 |
|  | 1ubi.A | 1ev9.A |  |  |  |  | 1.6 | 16 | 0.19 |
| * | 1uf4.A | 2e11.D | PF00795 |  | 6807 |  | 1.29 |  |  |
| * | 1ulu.A | 3sc6.D |  |  | 166 | NAD.NAP.0.916667 | 1.89 | 15 | 1.6 |
|  | 1umf.A | 3dli.C |  |  |  |  | 1.21 | 14 | 4.8 |
|  | 1uxz.A | 1grw.B |  |  |  |  | 1.54 |  |  |
| * | 1uyl.A | 1h7s.A | PF02518 |  | 5524 |  | 2.68 | 22 | 0.011 |
|  | 1v0s.A | 2g38.D |  |  |  |  | 0.96 |  |  |
| * | 1v6z.A | 1x7o.A |  |  | 8168 | SAH.SAM.0.962963 | 1.61 | 16 | 0.96 |
|  | 1vfj.A | 3bal.C |  |  |  |  | 0.95 |  |  |
|  | 1vic.A | 2ofz.A |  |  |  |  | 1.38 |  |  |
|  | 1vpn.A | 4dhi.B |  |  |  |  | 1.58 | 15 | 2.5 |
|  | 1w9a.A | 2j0v.A |  |  |  |  | 1.03 | 19 | 0.031 |
| * | 1wjg.A | 3cis.H | PF00582 |  | 166 | ACP.ATP.0.9375 | 2.28 | 23 | 0.002 |
| * | 1wny.A | 4arc.A |  |  | 166 |  | 1.54 | 27 | 6e-04 |
|  | 1wos.A | 2lme.B |  |  |  |  | 1.37 |  |  |
| * | 1ws9.A | 1w07.B | PF02770 |  | 3995 | FAD.FAD.1 | 1.94 | 27 | 0.001 |
|  | 1wtj.A | 3kvn.X |  |  |  |  | 1.2 | 18 | 0.65 |
| * | 1wxf.A | 3sdb.A |  |  | 166 | AMP.AMP.1 | 2.79 | 32 | 2e-05 |
| * | 1x56.A | 2ztg.A |  |  | 166 | 4AD.A5A.0.787879 | 2.27 | 25 | 0.01 |
| * | 1x7o.A | 1mxi.A | PF00588 |  | 3723 | SAM.SAH.0.962963 | 2.14 | 20 | 0.026 |
|  | 1xix.A | 3msw.A |  |  |  |  | 1.28 | 17 | 0.24 |
| * | 1xk7.A | 1mxh.D |  |  | 8152 |  | 1.62 | 17 | 1 |
|  | 1xo6.A | 1efp.C |  |  |  |  | 0.85 | 17 | 1.2 |
| * | 1xqo.A | 1o7n.B |  |  | 3824 |  | 1.13 | 13 | 4.7 |
|  | 1xw2.A | 3byb.B |  |  |  |  | 1.19 |  |  |
| * | 1y2q.A | 3ko5.D |  |  | 5737 | SSA.ADP.0.647059 | 1.36 |  |  |
|  | 1y2t.A | 2jen.A |  |  |  |  | 1.13 |  |  |
|  | 1ybt.A | 1vfr.B |  |  |  |  | 1.68 | 13 | 5.8 |
|  | 1yvr.A | 3fv6.B |  |  |  |  | 1.52 | 17 | 0.63 |
| * | 1yvy.B | 1khb.A |  |  | 166 | ATP.GCP.0.852941 | 2.21 | 17 | 3 |
| * | 1z7g.A | 1nh2.D |  |  | 5515 |  | 1.31 |  |  |
| * | 1zah.A | 2iqt.A | PF00274 | 4.1.2.13 | 3824 |  | 2.97 | 23 | 0.009 |
| * | 1zcu.A | 1ga8.A | PF01501 |  | 16740 | MN.MN.1 | 2.08 | 18 | 0.25 |
|  | 1znw.A | 3fvz.A |  |  |  |  | 1.28 | 17 | 0.52 |
|  | 1zty.A | 3ery.A |  |  |  |  | 0.86 | 17 | 0.42 |
| * | 1zuh.A | 3uie.B |  |  | 5524 | ADP.ADX.0.928571 | 2.07 |  |  |
|  | 2a6z.A | 3t58.D |  |  |  |  | 1.07 |  |  |
|  | 2a8f.A | 2yf2.D |  |  |  |  | 1.64 |  |  |
| * | 2ahf.A | 3pmm.A | PF07470 |  | 3824 |  | 1.79 | 21 | 0.06 |
| * | 2ahu.A | 2oas.A |  |  | 16740 | COA.COA.1 | 1.27 | 18 | 0.74 |
|  | 2amj.A | 2ov3.A |  |  |  |  | 1.45 |  |  |
| * | 2b0j.A | 3fi9.B |  |  | 16491 |  | 1.28 | 19 | 0.18 |
|  | 2b6p.A | 4tmk.A |  |  |  |  | 1.21 |  |  |
| * | 2b78.A | 2jjq.A |  |  | 8168 | SAH.SAH.1 | 1.95 | 25 | 0.004 |
| * | 2b98.A | 1hqk.D | PF00885 |  | 9231 |  | 1.96 | 28 | 2e-05 |
|  | 2bgt.A | 1jb3.A |  |  |  |  | 1.21 | 16 | 0.5 |
| * | 2bjw.A | 3d8b.B |  |  | 166 | ADP.ADP.1 | 2.19 | 13 | 7.7 |
|  | 2boe.X | 2j3x.A |  |  |  |  | 0.99 |  |  |
|  | 2c61.A | 4esu.A |  |  |  |  | 1.51 | 18 | 0.24 |
| * | 2c7i.A | 1w66.A | PF03099 |  | 3824 | LPA.DKA.0.714286 | 1.54 | 25 | 0.001 |
|  | 2car.A | 3rrc.A |  |  |  |  | 1.21 | 14 | 3.9 |
|  | 2chs.A | 2jdi.I |  |  |  |  | 1.39 | 14 | 0.2 |
| * | 2ci3.A | 1s9r.B | PF02274 |  | 5737 | CIR.ARG.0.846154 | 1.76 | 25 | 0.002 |
|  | 2cwk.A | 3uv4.B |  |  |  |  | 1.43 |  |  |
|  | 2cx5.A | 2qgu.A |  |  |  |  | 1.77 | 19 | 0.029 |
|  | 2d59.A | 2vhs.D |  |  |  |  | 1.51 | 15 | 0.87 |
|  | 2dhq.A | 3aaf.B |  |  |  |  | 1.68 | 20 | 0.007 |
| * | 2dj6.A | 3jyg.F | PF01242 |  |  |  | 2 | 26 | 9e-05 |
| * | 2dka.A | 3i3w.A | PF00408 |  | 287 | ZN.ZN.1 | 2.28 | 32 | 4e-05 |
| * | 2dps.A | 3p2l.E |  |  | 5737 |  | 1.28 | 19 | 0.08 |
|  | 2dqw.A | 3eeg.B |  |  |  |  | 1.17 | 19 | 0.11 |
| * | 2e0c.A | 1ptm.A |  |  | 287 |  | 2.22 | 20 | 0.063 |
| * | 2e0k.A | 3nut.A | PF00590 |  | 8152 | SAH.SAH.1 | 2.1 | 32 | 6e-06 |
|  | 2e10.A | 2ddz.C |  |  |  |  | 0.95 | 12 | 7.6 |
| * | 2e1v.A | 3fot.A |  |  | 16740 |  | 1.68 | 16 | 3.1 |
| * | 2ecr.A | 1usc.A | PF01613 |  | 10181 | NAD.NAP.0.916667 | 1.79 | 26 | 2e-04 |
|  | 2ex0.A | 2oaj.A |  |  |  |  | 1.03 | 17 | 1.7 |
|  | 2f82.A | 2nps.C |  |  |  |  | 1.67 | 15 | 0.72 |
|  | 2f9t.A | 2j5p.A |  |  |  |  | 1.27 |  |  |
| * | 2fk7.A | 2o57.D |  |  | 8168 |  | 2.05 | 29 | 1e-04 |
|  | 2fp8.A | 3at7.A |  |  |  |  | 1.03 | 14 | 4.5 |
| * | 2fsf.A | 4a15.A |  |  | 166 |  | 1.63 | 19 | 0.93 |
| * | 2g67.A | 2o1x.D |  |  | 3824 | MG.MG.1 | 2.36 | 18 | 1.7 |
| * | 2g95.A | 2im5.A | PF04095 |  | 4514 |  | 1.94 | 17 | 1.7 |
|  | 2gfv.A | 3sdb.A |  |  |  |  | 1.08 | 15 | 6 |
| * | 2gg4.A | 1dci.A |  |  | 3824 | SO4.SO4.1 | 1.05 | 19 | 0.2 |
|  | 2gqv.A | 2yuz.A |  |  |  |  | 1.55 |  |  |
| * | 2gsf.A | 1z57.A |  |  | 4672 |  | 1.94 | 30 | 7e-05 |
| * | 2gt2.A | 2o5f.A | PF00293 |  | 16787 |  | 1.64 | 27 | 5e-05 |
| * | 2gub.A | 3m0m.C | PF01261 |  | 16853 | GLC.AFD.1 | 2.59 | 19 | 0.24 |
| * | 2gwx.A | 3vhv.A | PF00104 |  | 3677 |  | 2.12 | 29 | 6e-05 |
| * | 2gyy.A | 2cvo.D | PF01118 |  | 3942 |  | 1.81 | 25 | 0.003 |
| * | 2h2z.A | 1lvm.A |  |  | 3824 |  | 1.9 | 15 | 1.4 |
| * | 2hbj.A | 2fby.A | PF01612 |  | 3676 | MN.MN.1 | 1.96 | 17 | 0.37 |
| * | 2hiv.A | 1fvi.A | PF01068 |  | 166 | ATP.AMP.0.741935 | 2.28 | 24 | 0.006 |
| * | 2hk0.A | 2qw5.A | PF01261 |  | 46872 |  | 2.28 | 27 | 6e-04 |
| * | 2hvm.A | 3n17.A |  |  | 3824 | NAA.NAG.1 | 2.5 | 24 | 0.004 |
|  | 2hy7.A | 2xbk.A |  |  |  |  | 0.99 | 19 | 0.19 |
| * | 2hzr.A | 1fm4.A |  |  |  | STR.DXC.0.758621 | 1.06 | 18 | 0.067 |
| * | 2i4l.A | 1b76.B | PF00587 |  | 166 | 5CA.GAP.0.806452 | 2.42 | 25 | 0.005 |
| * | 2iob.A | 4egq.B |  |  | 166 |  | 1.65 | 18 | 0.83 |
|  | 2j7v.A | 1w5d.A |  |  |  |  | 2.08 | 18 | 0.46 |
|  | 2j8n.A | 2xgf.B |  |  |  |  | 1.21 | 12 | 6.9 |
| * | 2jbr.A | 3gp0.A |  |  | 166 |  | 1.55 | 17 | 0.68 |
|  | 2nwd.X | 2y8p.B |  |  |  |  | 1.73 |  |  |
| * | 2nxc.A | 1wy7.C |  |  | 8168 | SAH.SAH.1 | 2.34 | 27 | 2e-04 |
| * | 2o9p.A | 2jep.B |  |  | 3824 |  | 2.03 | 15 | 6.9 |
| * | 2oam.A | 3nix.E |  |  | 166 | FAD.FAD.1 | 1.68 | 28 | 4e-04 |
| * | 2opt.A | 4g92.B |  |  | 3677 |  | 1.95 |  |  |
| * | 2ove.A | 1fdq.B | PF00061 |  |  | DAO.OLA.0.7 | 1.78 |  |  |
| * | 2paw.A | 2pqf.A | PF00644 |  | 3950 |  | 2.23 | 31 | 1e-05 |
| * | 2pfk.A | 3eg4.A |  |  | 5737 |  | 1.13 |  |  |
| * | 2pkf.A | 3ikh.B | PF00294 |  | 166 | ACP.ATP.0.9375 | 2.83 | 21 | 0.028 |
| * | 2q5r.A | 2fv7.A | PF00294 |  | 166 | ADP.ADP.1 | 1.71 | 32 | 1e-05 |
|  | 2q6z.A | 3lpp.A |  |  |  |  | 0.76 | 21 | 0.11 |
|  | 2qev.A | 1xhf.A |  |  |  |  | 1.08 | 17 | 0.064 |
| * | 2qsu.A | 4g41.A |  | 3.2.2.9 | 3824 | FMC.MTA.0.695652 | 1.92 | 31 | 1e-05 |
| * | 2qvl.A | 2cbp.A |  |  | 46872 |  | 1.37 |  |  |
| * | 2qys.A | 3h2s.B | PF05368 |  |  | NAP.NDP.1 | 1.8 | 27 | 3e-04 |
|  | 2r60.A | 3qve.C |  |  |  |  | 1.67 | 14 | 2.7 |
|  | 2rg7.A | 1wwv.A |  |  |  |  | 1.12 | 15 | 0.39 |
| * | 2rjd.A | 3m9q.A |  |  | 5634 |  | 1.93 | 25 | 2e-04 |
| * | 2sga.A | 2qxi.A | PF00089 |  | 3824 |  | 2.37 | 18 | 0.14 |
| * | 2sil.A | 4fzh.A |  |  | 4308 |  | 1.82 | 16 | 2.4 |
| * | 2ts1.A | 3p0j.C | PF00579 | 6.1.1.1 | 166 |  | 1.98 | 18 | 0.63 |
| * | 2uyo.A | 1rjd.A | PF04072 |  | 8168 | SAM.SAH.0.962963 | 1.83 |  |  |
| * | 2v78.A | 2pkf.B | PF00294 |  | 166 | AMP.2FA.0.791667 | 1.92 | 29 | 1e-04 |
|  | 2vfb.A | 3r0n.A |  |  |  |  | 1.24 | 17 | 0.18 |
| * | 2vfy.A | 1woj.A |  |  | 3824 |  | 2.01 | 16 | 0.47 |
|  | 2vq4.A | 3ixc.A |  |  |  |  | 1.11 | 12 | 3.6 |
|  | 2vua.A | 1f20.A |  |  |  |  | 1.26 |  |  |
| * | 2wn4.A | 2gwm.A |  |  | 5576 | NAD.NAD.1 | 1.94 | 24 | 0.003 |
| * | 2wvh.A | 2x7j.D | PF02775 |  | 3824 | PYR.EDO.0.666667 | 1.45 | 24 | 0.015 |
| * | 2wzt.A | 2c91.J | PF00248 |  | 16491 | NA7.NAP.0.8125 | 1.39 | 32 | 1e-05 |
| * | 2x5s.A | 3brk.X | PF00483 |  | 166 |  | 2.49 | 19 | 0.17 |
|  | 2ywb.A | 3gza.B |  |  |  |  | 0.94 |  |  |
|  | 2ywm.A | 2fbn.A |  |  |  |  | 1.65 | 13 | 3.2 |
| * | 2yxf.A | 1i1c.B | PF07654 |  |  |  | 1.69 | 32 | 5e-07 |
| * | 2yya.A | 3mwd.A |  |  | 5524 | ATP.ADP.0.870968 | 1.74 | 18 | 0.54 |
| * | 2yyt.A | 4dbe.B |  | 4.1.1.23 | 3824 | C5P.BMP.0.869565 | 1.9 | 27 | 2e-04 |
| * | 2yzg.A | 3orr.A |  |  | 166 | ADP.ADP.1 | 2.59 | 26 | 0.001 |
|  | 2zbs.A | 2q73.D |  |  |  |  | 1.8 |  |  |
| * | 2zcg.A | 4dbe.B |  | 4.1.1.23 | 3824 | OMP.BMP.0.84 | 2.37 | 32 | 8e-06 |
|  | 2zco.A | 2pjw.V |  |  |  |  | 1.68 | 18 | 0.038 |
| * | 2zgl.A | 2zhn.A | PF00337 |  | 30246 |  | 2.48 | 18 | 0.072 |
|  | 2zhy.B | 4iqn.B |  |  |  |  | 1.22 | 14 | 1.6 |
| * | 2zj8.A | 2d7d.A | PF00271 |  | 166 | ACP.ADP.0.8125 | 2.04 | 21 | 0.23 |
| * | 3a0y.A | 2e0a.A | PF02518 |  | 5524 | ADP.ADP.1 | 2.16 | 14 | 3.2 |
| * | 3a2r.X | 2xe1.A | PF00267 |  |  |  | 1.83 | 30 | 9e-05 |
| * | 3a5q.A | 4ewp.C |  |  | 3824 |  | 1.37 | 29 | 2e-04 |
| * | 3aap.A | 1u6z.A |  |  | 16787 |  | 1.61 | 22 | 0.049 |
| * | 3ado.A | 3b1f.A |  |  | 55114 | NAI.NAD.1 | 2.03 | 20 | 0.071 |
| * | 3app.A | 3qrv.A | PF00026 |  | 4190 |  | 2.21 | 32 | 1e-05 |
| * | 3b3g.A | 2yxd.A |  |  | 6479 |  | 1.83 | 29 | 5e-05 |
|  | 3ba1.A | 3bhg.A |  |  |  |  | 1.52 | 18 | 0.6 |
|  | 3blm.A | 2uwx.A |  |  |  |  | 1.81 | 15 | 2.7 |
| * | 3btv.A | 3dty.A | PF01408 |  | 16491 |  | 1.67 | 27 | 0.001 |
|  | 3bue.A | 3fge.A |  |  |  |  | 0.96 | 13 | 2.1 |
| * | 3byl.A | 1st8.A |  |  |  | RAF.SUC.0.676471 | 1.39 | 20 | 0.23 |
|  | 3c2e.A | 2p2r.A |  |  |  |  | 1.05 |  |  |
| * | 3c8n.A | 1y6j.A |  |  | 5975 |  | 0.99 | 14 | 4.5 |
|  | 3caf.A | 2x8n.A |  |  |  |  | 1.27 |  |  |
|  | 3cb6.A | 3n6m.A |  |  |  |  | 1.35 | 15 | 5.7 |
|  | 3cgz.A | 1shm.A |  |  |  |  | 0.89 | 13 | 1.8 |
|  | 3cou.A | 2obs.A |  |  |  |  | 1.33 | 14 | 3.5 |
|  | 3cq1.A | 3mkl.B |  |  |  |  | 1.26 | 18 | 0.024 |
| * | 3crm.A | 2ze6.A |  |  | 16740 | CL.CL.1 | 2.6 | 28 | 1e-04 |
| * | 3csr.A | 3cql.A |  |  |  | NAG.NAG.1 | 0.92 | 22 | 0.003 |
|  | 3ctb.A | 2ppi.A |  |  |  |  | 1.72 | 21 | 0.014 |
| * | 3d0o.A | 3awd.B |  |  | 16491 |  | 2.21 | 17 | 0.38 |
|  | 3d95.A | 3bb9.D |  |  |  |  | 1.58 | 15 | 0.64 |
|  | 3drd.A | 2lty.A |  |  |  |  | 1.32 |  |  |
| * | 3dre.A | 1r62.A |  |  | 5524 |  | 1.49 | 18 | 0.18 |
| * | 3dul.A | 3sso.B |  |  | 8168 | SAH.SAH.1 | 1.59 | 20 | 0.041 |
| * | 3e1s.A | 4b3f.X |  |  | 166 |  | 1.74 | 25 | 0.009 |
| * | 3e5b.A | 2hjp.A | PF00463 |  | 3824 |  | 1.65 | 32 | 1e-05 |
|  | 3eiz.A | 1em2.A |  |  |  |  | 1.21 | 15 | 1.6 |
| * | 3ek6.A | 3asz.A |  |  | 166 |  | 1.94 | 20 | 0.039 |
|  | 3etf.A | 3dcm.X |  |  |  |  | 1.62 | 16 | 1.4 |
|  | 3evh.A | 3e38.A |  |  |  |  | 1.33 | 17 | 0.7 |
|  | 3ex9.A | 1jmk.C |  |  |  |  | 1.36 |  |  |
| * | 3exr.A | 1eix.A | PF00215 |  | 3824 |  | 1.81 | 19 | 0.064 |
| * | 3f1l.A | 1wma.A | PF00106 |  | 8152 | NAP.NAP.1 | 2.8 | 31 | 1e-05 |
| * | 3f6f.A | 3n5o.A |  | 2.5.1.18 | 4364 |  | 1.41 | 25 | 5e-04 |
|  | 3ftd.A | 1lss.B |  |  |  |  | 1.82 | 18 | 0.092 |
| * | 3fv6.A | 2qrd.G | PF00571 |  | 3824 | ADP.ADP.1 | 2.16 | 23 | 0.003 |
| * | 3g1s.A | 3g3d.A | PF00215 |  | 3824 | 5FU.16B.0.791667 | 1.84 | 30 | 2e-05 |
|  | 3gbt.A | 4gkw.B |  |  |  |  | 1.34 |  |  |
|  | 3gd0.A | 2i0w.A |  |  |  |  | 1.95 | 15 | 2.8 |
| * | 3gdk.A | 3ve9.B |  | 4.1.1.23 | 3824 | UP6.BMP.0.869565 | 2.53 | 24 | 0.002 |
| * | 3glk.A | 2e74.A |  |  | 46872 |  | 1.55 | 20 | 0.054 |
| * | 3gpg.A | 2acf.B | PF01661 |  |  | APR.APR.1 | 2.57 |  |  |
|  | 3gqh.A | 3sfx.A |  |  |  |  | 1.38 | 24 | 0.001 |
|  | 3gsz.A | 3mpz.D |  |  |  |  | 1.05 |  |  |
| * | 3gva.A | 3hn6.F |  |  | 3824 |  | 1.68 | 13 | 2.7 |
|  | 3h2g.A | 1g66.A |  |  |  |  | 1.64 | 22 | 0.009 |
|  | 3h38.A | 2yqy.B |  |  |  |  | 1.49 | 16 | 0.76 |
|  | 3h49.A | 2di9.A |  |  |  |  | 1.42 | 13 | 3.3 |
| * | 3h71.A | 4fzh.A |  |  | 4308 |  | 1.91 | 20 | 0.14 |
|  | 3hbh.A | 3ouz.B |  |  |  |  | 0.9 | 17 | 0.57 |
| * | 3his.A | 2b7u.A | PF00161 |  | 17148 |  | 2.47 | 17 | 0.38 |
| * | 3hj4.A | 2q0d.B |  |  |  | UTP.ATP.0.714286 | 2.18 | 16 | 1.6 |
|  | 3hnx.A | 1h4g.A |  |  |  |  | 1.49 | 18 | 0.056 |
| * | 3i0c.A | 1ga8.A |  |  |  | MN.MN.1 | 1.53 | 28 | 1e-04 |
| * | 3i3i.A | 2wg9.B |  |  | 4623 |  | 1.49 | 27 | 2e-05 |
| * | 3i8s.A | 4bas.A |  |  | 5525 | GCP.GNP.0.939394 | 2.53 | 26 | 3e-04 |
| * | 3ik8.A | 2kgj.A | PF00639 |  | 16853 |  | 1.13 | 25 | 1e-04 |
|  | 3ily.A | 3mj6.A |  |  |  |  | 0.64 |  |  |
| * | 3ity.A | 1d8w.C |  |  | 16853 | AOS.RNS.0.916667 | 2.56 | 30 | 1e-04 |
|  | 3iuj.A | 1fdq.B |  |  |  |  | 1.2 |  |  |
| * | 3jyl.A | 3nx4.A | PF00107 |  | 166 | NDP.NAP.1 | 1.28 | 25 | 0.002 |
|  | 3k0m.A | 3syj.A |  |  |  |  | 1.32 | 15 | 3.6 |
| * | 3k5o.A | 2py5.A |  | 2.7.7.7 | 166 | DCP.DGT.0.735294 | 2.42 | 20 | 0.34 |
| * | 3kaj.A | 3n6x.A |  |  |  | SO4.SO4.1 | 1.65 | 18 | 1.1 |
|  | 3kdh.A | 3k04.B |  |  |  |  | 1.92 | 17 | 0.12 |
| * | 3kje.A | 1ion.A | PF01656 |  | 166 | ADP.ADP.1 | 1.56 | 22 | 0.012 |
|  | 3kjt.A | 3cw9.B |  |  |  |  | 0.92 | 15 | 3.6 |
|  | 3kp7.A | 2ewd.A |  |  |  |  | 2.18 | 17 | 0.28 |
| * | 3kr9.A | 2pbf.B |  |  | 8168 | SAM.SAH.0.962963 | 1.6 | 18 | 0.12 |
|  | 3kx7.A | 2q78.C |  |  |  |  | 1.73 | 20 | 0.01 |
| * | 3lig.A | 3ugf.B |  |  | 4553 | DQR.DQR.1 | 2.16 | 21 | 0.13 |
|  | 3loi.A | 3d82.A |  |  |  |  | 1.79 |  |  |
|  | 3m4d.A | 1zxj.C |  |  |  |  | 1.17 | 17 | 0.5 |
| * | 3n6j.A | 3sbf.B |  |  | 3824 | GKR.D8T.0.785714 | 1.44 | 17 | 1.4 |
| * | 3nk6.A | 1mxi.A | PF00588 |  | 1510 | SAM.SAH.0.962963 | 2.27 | 30 | 1e-05 |
| * | 3pte.A | 3vsk.A |  |  |  | CEF.CEF.1 | 1.53 |  |  |
| * | 4ake.A | 2plr.A |  |  | 166 |  | 1.83 | 15 | 1.2 |
| * | 4pgm.A | 3mxo.A | PF00300 |  |  |  | 1.96 | 21 | 0.011 |
| * | 4pti.A | 3m7q.B | PF00014 |  | 4867 |  | 2.8 | 31 | 4e-08 |

Table S3. Validation of binding site comparison for function prediction on a set of 369 benchmark PDB protein structures with known functions using BLAST sequence alignment tool. Asterisk (*) in the first column indicates that function of the benchmark protein was correctly predicted, i.e., the benchmark and the similar protein have at least one function identifier (see Pfam, EC, GO, and Ligand columns) in common.

| Funct.  Match | Benchmark PDB.CHAIN | Similar PDB.CHAIN | Pfam number | EC number | GO term | Ligand  #1.#2.Tanimoto | BLAST  bit-score | BLAST  expect. |  |
| --- | --- | --- | --- | --- | --- | --- | --- | --- | --- |
|  | 180l.A | 1xm9.A |  |  |  |  | 28 | 1e-04 |  |
| * | 1a4u.A | 3uwr.D | PF00106 |  | 8152 | NAD.NAD.1 | 32 | 5e-06 |  |
| * | 1ade.A | 1dwl.B |  |  | 46872 |  | 28 | 1e-05 |  |
|  | 1ak1.A | 3tbf.G |  |  |  |  | 31 | 2e-05 |  |
|  | 1akz.A | 2zb4.A |  |  |  |  | 30 | 2e-05 |  |
|  | 1arb.A | 2o01.3 |  |  |  |  | 31 | 5e-06 |  |
|  | 1arl.A | 2k1s.A |  |  |  |  | 28 | 5e-05 |  |
|  | 1az3.A | 3egn.A |  |  |  |  | 28 | 3e-05 |  |
|  | 1az5.A | 2xzn.S |  |  |  |  | 32 | 3e-07 |  |
| * | 1b8e.A | 1dzk.B | PF00061 |  | 5576 | DAO.UNA.0.857143 | 32 | 9e-07 |  |
|  | 1b8p.A | 1oz9.A |  |  |  |  | 31 | 7e-06 |  |
| * | 1bd9.A | 3pxx.E |  |  | 166 |  | 31 | 8e-06 |  |
|  | 1bec.A | 3qq4.B |  |  |  |  | 32 | 5e-07 |  |
| * | 1bk7.A | 4dw5.B |  |  | 3723 | U5P.C.0.909091 | 28 | 3e-05 |  |
| * | 1bkz.A | 2wkk.C | PF00337 |  | 5576 | GAL.A2G.0.6875 | 30 | 2e-06 |  |
| * | 1bqc.A | 4hty.A |  |  | 3824 |  | 32 | 2e-05 |  |
| * | 1byi.A | 1ion.A | PF01656 |  | 166 | ACP.ADP.0.8125 | 32 | 3e-06 |  |
|  | 1c48.B | 3frx.B |  |  |  |  | 26 | 7e-05 |  |
| * | 1c5h.A | 3p3g.A |  |  | 16787 |  | 29 | 4e-05 |  |
| * | 1ceo.A | 3oq2.B |  |  | 16787 |  | 29 | 1e-05 |  |
| * | 1cex.A | 2czq.A | PF01083 |  | 8152 |  | 28 | 6e-05 |  |
|  | 1cpj.A | 2c21.A |  |  |  |  | 28 | 5e-05 |  |
| * | 1crw.G | 3aog.G |  |  | 16491 | SO4.PO4.0.666667 | 32 | 2e-05 |  |
| * | 1cwy.A | 2wl9.A |  |  | 3824 | EDO.GOL.0.666667 | 32 | 2e-05 |  |
|  | 1dco.A | 2bib.A |  |  |  |  | 29 | 2e-05 |  |
|  | 1dhn.A | 2jjs.D |  |  |  |  | 29 | 4e-06 |  |
|  | 1dq0.A | 1uti.A |  |  |  |  | 25 | 3e-05 |  |
|  | 1dup.A | 3gnf.B |  |  |  |  | 29 | 3e-05 |  |
|  | 1e4f.T | 4e2h.A |  |  |  |  | 31 | 2e-05 |  |
|  | 1e5l.A | 1ue1.B |  |  |  |  | 30 | 3e-05 |  |
|  | 1e8y.A | 3llh.B |  |  |  |  | 28 | 7e-05 |  |
| * | 1edq.A | 3n17.A |  |  | 3824 | NAA.NAG.1 | 30 | 7e-05 |  |
| * | 1epa.A | 1exs.A | PF00061 |  | 5576 |  | 31 | 3e-06 |  |
| * | 1ewz.A | 1vqq.B | PF00905 |  | 8658 |  | 32 | 1e-05 |  |
|  | 1ey0.A | 2iq1.A |  |  |  |  | 27 | 8e-05 |  |
| * | 1f14.A | 1reo.A |  |  | 16491 |  | 32 | 2e-05 |  |
|  | 1f1s.A | 1l3k.A |  |  |  |  | 30 | 5e-05 |  |
|  | 1f2v.A | 1eyb.A |  |  |  |  | 31 | 2e-05 |  |
| * | 1f41.A | 2h0e.B | PF00576 |  | 6810 |  | 30 | 1e-06 |  |
| * | 1f5z.A | 3e96.A | PF00701 |  | 3824 |  | 32 | 1e-05 |  |
|  | 1fcq.A | 2a4x.A |  |  |  |  | 29 | 2e-05 |  |
|  | 1fgb.D | 3gbe.A |  |  |  |  | 30 | 7e-06 |  |
|  | 1fo9.A | 1a8q.A |  |  |  |  | 29 | 9e-05 |  |
| * | 1fsf.A | 1y89.B | PF01182 |  | 5975 |  | 32 | 8e-06 |  |
| * | 1ftf.B | 2c43.A | PF01648 |  | 287 |  | 32 | 2e-06 |  |
| * | 1ftr.A | 1cbi.B |  |  | 5737 |  | 28 | 3e-05 |  |
|  | 1fwl.A | 2mob.A |  |  |  |  | 27 | 9e-05 |  |
|  | 1g40.A | 4ayi.E |  |  |  |  | 32 | 1e-06 |  |
| * | 1g4e.B | 1xm3.B |  |  | 3824 |  | 32 | 5e-06 |  |
|  | 1g95.A | 3tio.F |  |  |  |  | 32 | 6e-06 |  |
|  | 1gbs.A | 1t6a.A |  |  |  |  | 28 | 2e-05 |  |
|  | 1gce.A | 3mvn.A |  |  |  |  | 31 | 1e-05 |  |
| * | 1gfs.A | 1aoe.B |  |  | 16491 | NAP.NAP.1 | 31 | 8e-06 |  |
| * | 1gou.A | 1lni.B | PF00545 |  | 3723 | 3GP.2GP.0.714286 | 28 | 7e-06 |  |
|  | 1gsh.A | 3ejv.A |  |  |  |  | 31 | 1e-05 |  |
|  | 1gwk.A | 3lvu.A |  |  |  |  | 28 | 3e-05 |  |
|  | 1gy0.A | 3zzl.C |  |  |  |  | 28 | 4e-06 |  |
|  | 1hk9.A | 1ycy.A |  |  |  |  | 25 | 1e-05 |  |
| * | 1hka.A | 3p0y.A |  |  | 5524 |  | 27 | 9e-05 |  |
| * | 1ho1.A | 1gkz.A |  |  | 16740 |  | 29 | 1e-04 |  |
|  | 1i7n.A | 3bld.A |  |  |  |  | 31 | 3e-05 |  |
|  | 1iad.A | 1m2s.A |  |  |  |  | 25 | 2e-05 |  |
|  | 1ilv.A | 1zav.A |  |  |  |  | 30 | 9e-06 |  |
|  | 1inl.A | 2db8.A |  |  |  |  | 28 | 2e-05 |  |
| * | 1j85.A | 1gz0.B | PF00588 |  | 3723 |  | 30 | 8e-06 |  |
| * | 1j8s.A | 1hlc.A |  |  | 30246 | BGC.BGC.1 | 26 | 9e-05 |  |
|  | 1jcf.A | 3ftd.A |  |  |  |  | 30 | 3e-05 |  |
|  | 1jks.A | 1neg.A |  |  |  |  | 30 | 4e-06 |  |
|  | 1jxo.A | 2kro.A |  |  |  |  | 32 | 2e-07 |  |
|  | 1jyk.A | 2lmc.B |  |  |  |  | 29 | 4e-06 |  |
|  | 1k0m.A | 1aw9.A |  |  |  |  | 32 | 3e-06 |  |
| * | 1k3o.A | 3ein.A | PF00043 | 2.5.1.18 | 4364 | GSB.GTT.0.740741 | 32 | 3e-06 |  |
|  | 1k6a.A | 1d4x.A |  |  |  |  | 31 | 2e-05 |  |
| * | 1kam.A | 3nv7.A | PF01467 |  | 166 | DND.COA.0.614035 | 30 | 5e-06 |  |
| * | 1kf5.A | 1m07.A | PF00074 |  | 3676 | PO4.PO4.1 | 31 | 8e-07 |  |
|  | 1khd.B | 1u7k.B |  |  |  |  | 29 | 3e-05 |  |
|  | 1kn9.A | 3u5e.S |  |  |  |  | 31 | 6e-06 |  |
| * | 1kpa.A | 1fit.A | PF01230 |  | 166 | ADW.A.0.785714 | 32 | 5e-07 |  |
| * | 1kwb.B | 1h97.B |  |  | 46872 |  | 30 | 1e-05 |  |
| * | 1l7d.A | 2d1y.C |  |  | 166 | APR.NAD.0.777778 | 31 | 2e-05 |  |
| * | 1lbv.A | 2q74.A | PF00459 | 3.1.3.25 | 8934 |  | 32 | 6e-06 |  |
| * | 1lci.A | 4gs5.A |  |  | 3824 |  | 32 | 2e-05 |  |
|  | 1lf4.A | 3i08.B |  |  |  |  | 26 | 6e-05 |  |
|  | 1ltu.A | 2otk.F |  |  |  |  | 26 | 5e-05 |  |
|  | 1m1z.A | 1d8k.A |  |  |  |  | 28 | 1e-05 |  |
| * | 1mkb.A | 3d6x.C | PF07977 | 4.2.1.59 | 5737 |  | 30 | 3e-06 |  |
| * | 1mmi.A | 2xig.B |  |  | 3677 |  | 29 | 3e-05 |  |
| * | 1mr7.A | 3gvd.L |  |  | 16740 |  | 32 | 4e-06 |  |
| * | 1mtz.A | 1a88.A | PF00561 |  |  |  | 32 | 9e-06 |  |
|  | 1mwk.A | 1ks5.A |  |  |  |  | 30 | 3e-05 |  |
|  | 1mzl.A | 2pby.C |  |  |  |  | 24 | 4e-04 |  |
|  | 1n05.A | 1qyn.A |  |  |  |  | 26 | 1e-04 |  |
|  | 1ndb.A | 4i6l.B |  |  |  |  | 27 | 4e-05 |  |
|  | 1noa.A | 2hj3.A |  |  |  |  | 27 | 3e-05 |  |
| * | 1non.A | 3o7m.C | PF00156 |  | 9116 |  | 32 | 2e-06 |  |
|  | 1nxm.A | 2yzi.B |  |  |  |  | 27 | 5e-05 |  |
|  | 1o24.A | 1xo8.A |  |  |  |  | 30 | 7e-06 |  |
| * | 1oem.X | 1m3g.A |  |  | 6470 |  | 32 | 2e-06 |  |
|  | 1ofp.A | 2bzw.B |  |  |  |  | 26 | 8e-06 |  |
| * | 1ogh.A | 2yzj.A | PF00692 |  | 16787 | DCP.DUD.0.793103 | 32 | 2e-06 |  |
|  | 1ogl.A | 1u3e.M |  |  |  |  | 30 | 1e-05 |  |
| * | 1ogm.X | 1uu1.D |  |  | 8152 |  | 30 | 9e-05 |  |
|  | 1ojq.A | 3nau.A |  |  |  |  | 27 | 1e-05 |  |
| * | 1ooi.X | 3k1e.B | PF01395 |  | 5549 |  | 30 | 3e-06 |  |
|  | 1opy.A | 2rfr.A |  |  |  |  | 32 | 7e-07 |  |
| * | 1oxt.A | 1uf9.B |  |  | 166 | ADP.ATP.0.870968 | 32 | 6e-06 |  |
| * | 1p1x.A | 2wkj.D |  |  | 3824 | HPD.LAI.0.642857 | 30 | 3e-05 |  |
| * | 1p5h.A | 3amt.A |  |  | 5737 |  | 32 | 2e-05 |  |
|  | 1p6z.R | 4atq.E |  |  |  |  | 27 | 7e-05 |  |
|  | 1p74.A | 2a9v.D |  |  |  |  | 31 | 8e-06 |  |
|  | 1pdb.A | 2h8e.A |  |  |  |  | 28 | 3e-05 |  |
|  | 1png.A | 1x94.A |  |  |  |  | 31 | 1e-05 |  |
| * | 1q52.A | 3q1t.A | PF00378 |  | 3824 |  | 32 | 1e-05 |  |
|  | 1q7m.A | 1rmv.A |  |  |  |  | 30 | 4e-05 |  |
|  | 1qcz.A | 3cvj.A |  |  |  |  | 28 | 5e-05 |  |
|  | 1qid.A | 1g60.B |  |  |  |  | 31 | 3e-05 |  |
|  | 1qto.A | 2rk9.B |  |  |  |  | 28 | 1e-05 |  |
| * | 1qtr.A | 2yys.A | PF00561 |  | 6508 |  | 32 | 9e-06 |  |
| * | 1r0m.A | 4h1z.E |  |  | 3824 | MG.MG.1 | 32 | 1e-05 |  |
|  | 1r12.A | 4dpp.A |  |  |  |  | 31 | 2e-05 |  |
|  | 1r29.A | 2l9d.A |  |  |  |  | 28 | 7e-06 |  |
|  | 1rgp.A | 3cx3.A |  |  |  |  | 29 | 4e-05 |  |
| * | 1ri5.A | 1wzn.B |  |  | 8168 | SA8.SAH.0.892857 | 30 | 2e-05 |  |
| * | 1riq.A | 2z43.C |  |  | 166 |  | 31 | 3e-05 |  |
|  | 1rj1.A | 2cv6.A |  |  |  |  | 29 | 3e-05 |  |
|  | 1rkm.A | 3mfx.B |  |  |  |  | 28 | 6e-05 |  |
|  | 1rzv.A | 1mhm.B |  |  |  |  | 29 | 8e-06 |  |
|  | 1s2l.A | 1t5j.A |  |  |  |  | 27 | 1e-04 |  |
| * | 1s7k.A | 2i79.B | PF00583 |  | 8080 | COA.ACO.0.941176 | 32 | 2e-06 |  |
|  | 1sgk.A | 2x1p.D |  |  |  |  | 29 | 3e-05 |  |
| * | 1sgz.A | 3vav.D |  |  |  | GOL.EDO.0.666667 | 32 | 1e-05 |  |
| * | 1sjy.A | 1ktg.B | PF00293 |  | 166 | ATP.AMP.0.741935 | 32 | 6e-07 |  |
|  | 1snt.A | 2isa.B |  |  |  |  | 32 | 3e-05 |  |
|  | 1sqg.A | 2uwq.A |  |  |  |  | 27 | 6e-05 |  |
| * | 1sul.A | 2p5s.B |  |  | 5525 | GDP.GDP.1 | 32 | 3e-06 |  |
| * | 1swh.B | 1wbi.D | PF01382 |  |  | BTN.BTN.1 | 30 | 3e-06 |  |
|  | 1tgn.A | 4h5i.A |  |  |  |  | 30 | 5e-05 |  |
|  | 1thv.A | 2hrv.A |  |  |  |  | 28 | 2e-05 |  |
| * | 1tib.A | 3pe6.A |  |  | 4091 |  | 32 | 9e-06 |  |
| * | 1tje.A | 2kmm.A | PF02824 |  |  |  | 30 | 2e-06 |  |
|  | 1tjv.A | 2nml.A |  |  |  |  | 29 | 1e-05 |  |
| * | 1tvn.A | 2jep.B |  |  | 3824 |  | 30 | 5e-05 |  |
|  | 1tw0.A | 3kl1.B |  |  |  |  | 32 | 2e-06 |  |
| * | 1tyf.A | 3rst.H |  |  | 6508 |  | 32 | 4e-06 |  |
|  | 1tyv.A | 3gnl.B |  |  |  |  | 32 | 1e-05 |  |
|  | 1tzv.A | 3cx5.A |  |  |  |  | 30 | 1e-05 |  |
|  | 1u09.A | 1zpv.C |  |  |  |  | 27 | 6e-05 |  |
| * | 1u0t.A | 1cyg.A |  |  | 16740 |  | 32 | 3e-05 |  |
| * | 1u7u.A | 1p9o.B | PF04127 |  | 4632 |  | 30 | 3e-05 |  |
|  | 1uaj.A | 2hwv.A |  |  |  |  | 32 | 2e-06 |  |
|  | 1ubi.A | 1we6.A |  |  |  |  | 32 | 7e-08 |  |
|  | 1uf4.A | 3gra.A |  |  |  |  | 32 | 8e-06 |  |
| * | 1ulu.A | 3sx2.C | PF00106 |  | 8152 | NAD.NAD.1 | 32 | 8e-06 |  |
|  | 1umf.A | 2vu1.B |  |  |  |  | 30 | 5e-05 |  |
| * | 1uxz.A | 1od3.A | PF03422 |  | 30246 | BGC.BGC.1 | 31 | 2e-06 |  |
|  | 1uyl.A | 4ag6.A |  |  |  |  | 29 | 8e-05 |  |
|  | 1v0s.A | 3k3c.B |  |  |  |  | 30 | 2e-05 |  |
|  | 1v6z.A | 2bpo.A |  |  |  |  | 29 | 1e-04 |  |
|  | 1vfj.A | 3m05.A |  |  |  |  | 26 | 5e-05 |  |
| * | 1vic.A | 4fce.A |  |  | 5737 |  | 32 | 2e-05 |  |
|  | 1vpn.A | 1sky.E |  |  |  |  | 30 | 7e-05 |  |
| * | 1w9a.A | 3f7e.B | PF01243 |  | 4733 |  | 32 | 7e-07 |  |
| * | 1wjg.A | 1mjh.A | PF00582 |  | 166 | ACP.ATP.0.9375 | 32 | 6e-07 |  |
|  | 1wny.A | 4aez.H |  |  |  |  | 31 | 4e-06 |  |
|  | 1wos.A | 2dak.A |  |  |  |  | 27 | 2e-05 |  |
|  | 1ws9.A | 2vyi.B |  |  |  |  | 28 | 6e-05 |  |
|  | 1wtj.A | 1ti3.A |  |  |  |  | 27 | 8e-05 |  |
| * | 1wxf.A | 3sdb.A |  |  | 166 | AMP.AMP.1 | 32 | 2e-05 |  |
| * | 1x56.A | 2bog.X |  |  |  | PO4.SO4.0.666667 | 30 | 6e-05 |  |
| * | 1x7o.A | 3kty.B | PF00588 |  | 3723 |  | 30 | 1e-05 |  |
|  | 1xix.A | 2fel.C |  |  |  |  | 31 | 2e-05 |  |
|  | 1xk7.A | 4b0m.A |  |  |  |  | 28 | 8e-05 |  |
|  | 1xo6.A | 1ef5.A |  |  |  |  | 30 | 8e-06 |  |
|  | 1xqo.A | 1ccd.A |  |  |  |  | 27 | 1e-05 |  |
| * | 1xw2.A | 2vuj.A |  |  | 4553 | XYP.XYP.1 | 31 | 2e-05 |  |
|  | 1y2q.A | 2p3y.A |  |  |  |  | 30 | 1e-05 |  |
| * | 1y2t.A | 3mm5.E |  |  |  | SER.GOL.0.625 | 28 | 7e-05 |  |
| * | 1ybt.A | 1fx2.A | PF00211 |  | 9190 |  | 32 | 3e-06 |  |
|  | 1yvr.A | 3ia4.B |  |  |  |  | 29 | 5e-05 |  |
|  | 1yvy.B | 1ae1.A |  |  |  |  | 30 | 5e-05 |  |
| * | 1z7g.A | 2xbu.A | PF00156 | 2.4.2.8 | 166 | 25H.5GP.0.62963 | 32 | 4e-06 |  |
|  | 1zah.A | 2vuw.A |  |  |  |  | 32 | 1e-05 |  |
|  | 1zcu.A | 3b09.A |  |  |  |  | 31 | 1e-06 |  |
| * | 1znw.A | 1qhx.A |  |  | 166 | 5GP.AGS.0.666667 | 31 | 4e-06 |  |
| * | 1zty.A | 3pam.A | PF00496 |  | 5215 |  | 30 | 4e-05 |  |
| * | 1zuh.A | 1knq.B | PF01202 |  | 166 | ADP.ACP.0.8125 | 30 | 6e-06 |  |
|  | 2a6z.A | 2bpa.2 |  |  |  |  | 30 | 1e-05 |  |
|  | 2a8f.A | 3tcm.A |  |  |  |  | 30 | 5e-06 |  |
| * | 2ahf.A | 3eei.B |  |  | 3824 |  | 31 | 2e-05 |  |
|  | 2ahu.A | 2pnl.B |  |  |  |  | 30 | 6e-05 |  |
| * | 2amj.A | 2hpv.C | PF02525 |  |  |  | 32 | 2e-06 |  |
|  | 2b0j.A | 1vsw.H |  |  |  |  | 29 | 4e-05 |  |
|  | 2b6p.A | 1vgy.A |  |  |  |  | 30 | 7e-05 |  |
|  | 2b78.A | 1re0.B |  |  |  |  | 31 | 1e-05 |  |
| * | 2b98.A | 1rvv.1 | PF00885 |  | 9231 | RDL.INI.0.76 | 28 | 1e-05 |  |
| * | 2bgt.A | 3shx.A |  |  |  | MG.MG.1 | 30 | 1e-05 |  |
|  | 2bjw.A | 3sng.A |  |  |  |  | 30 | 2e-05 |  |
|  | 2boe.X | 1xn7.A |  |  |  |  | 28 | 1e-05 |  |
|  | 2c61.A | 1lk2.B |  |  |  |  | 27 | 9e-05 |  |
| * | 2c7i.A | 2qhs.A | PF03099 |  | 3824 | LPA.OC9.0.75 | 32 | 5e-06 |  |
|  | 2car.A | 4dka.D |  |  |  |  | 27 | 3e-05 |  |
|  | 2chs.A | 1xiw.C |  |  |  |  | 25 | 6e-05 |  |
|  | 2ci3.A | 3hgu.B |  |  |  |  | 30 | 6e-05 |  |
|  | 2cwk.A | 1ksk.A |  |  |  |  | 28 | 6e-05 |  |
|  | 2cx5.A | 3gax.B |  |  |  |  | 30 | 3e-06 |  |
|  | 2d59.A | 4egu.B |  |  |  |  | 28 | 7e-06 |  |
| * | 2dhq.A | 1rpn.C |  |  | 16829 |  | 29 | 3e-05 |  |
|  | 2dj6.A | 1oed.B |  |  |  |  | 27 | 4e-05 |  |
| * | 2dka.A | 3f4w.B |  |  | 5975 |  | 32 | 1e-05 |  |
|  | 2dps.A | 2q5z.A |  |  |  |  | 32 | 7e-07 |  |
| * | 2dqw.A | 3k13.A | PF00809 |  | 42558 |  | 30 | 3e-05 |  |
|  | 2e0c.A | 1g3k.C |  |  |  |  | 31 | 1e-05 |  |
| * | 2e0k.A | 3nut.A | PF00590 |  | 8152 | SAH.SAH.1 | 32 | 6e-06 |  |
|  | 2e10.A | 2fdb.N |  |  |  |  | 27 | 8e-05 |  |
|  | 2e1v.A | 3idw.A |  |  |  |  | 28 | 1e-05 |  |
|  | 2ecr.A | 3tma.A |  |  |  |  | 30 | 2e-05 |  |
|  | 2ex0.A | 3pwx.B |  |  |  |  | 29 | 9e-05 |  |
| * | 2f82.A | 1mzj.B |  |  | 3824 | HMG.COA.0.827586 | 32 | 2e-05 |  |
|  | 2f9t.A | 2v4h.B |  |  |  |  | 32 | 1e-06 |  |
|  | 2fk7.A | 1umq.A |  |  |  |  | 32 | 6e-07 |  |
|  | 2fp8.A | 1cc3.B |  |  |  |  | 29 | 2e-05 |  |
|  | 2fsf.A | 2p08.B |  |  |  |  | 32 | 5e-06 |  |
| * | 2g67.A | 3vsj.D |  |  | 16491 |  | 32 | 3e-05 |  |
| * | 2g95.A | 3aqq.D |  |  | 5737 |  | 30 | 3e-05 |  |
| * | 2gfv.A | 2pz0.B |  |  | 6629 |  | 31 | 2e-05 |  |
|  | 2gg4.A | 1ifw.A |  |  |  |  | 27 | 4e-05 |  |
|  | 2gqv.A | 4i78.D |  |  |  |  | 28 | 4e-06 |  |
|  | 2gsf.A | 1b4f.B |  |  |  |  | 31 | 1e-06 |  |
| * | 2gt2.A | 3exq.A | PF00293 |  | 16787 |  | 32 | 7e-07 |  |
|  | 2gub.A | 3pa6.B |  |  |  |  | 30 | 6e-06 |  |
| * | 2gwx.A | 3k22.A | PF00104 |  | 3677 | B7G.BOG.0.95 | 32 | 6e-06 |  |
| * | 2gyy.A | 2g17.A | PF01118 |  | 3942 |  | 32 | 1e-05 |  |
|  | 2h2z.A | 3k31.B |  |  |  |  | 30 | 6e-05 |  |
|  | 2hbj.A | 3mso.A |  |  |  |  | 30 | 2e-05 |  |
|  | 2hiv.A | 3gxh.B |  |  |  |  | 32 | 8e-06 |  |
|  | 2hk0.A | 1njk.A |  |  |  |  | 30 | 1e-05 |  |
| * | 2hvm.A | 2y8v.C | PF00704 | 3.2.1.14 | 3824 |  | 30 | 3e-05 |  |
| * | 2hy7.A | 2dgk.E |  |  | 16020 |  | 31 | 5e-05 |  |
| * | 2hzr.A | 3o22.A | PF00061 |  | 5215 |  | 31 | 3e-06 |  |
|  | 2i4l.A | 1umq.A |  |  |  |  | 30 | 4e-06 |  |
|  | 2iob.A | 4aow.B |  |  |  |  | 32 | 2e-05 |  |
|  | 2j7v.A | 3a3j.A |  |  |  |  | 32 | 1e-05 |  |
| * | 2j8n.A | 1mk4.B | PF00583 |  | 8080 |  | 31 | 3e-06 |  |
|  | 2jbr.A | 1dbw.B |  |  |  |  | 28 | 4e-05 |  |
|  | 2nwd.X | 4b9i.A |  |  |  |  | 28 | 2e-05 |  |
| * | 2nxc.A | 1i1n.A |  |  | 5737 | SAH.SAH.1 | 32 | 4e-06 |  |
|  | 2o9p.A | 3f1y.C |  |  |  |  | 32 | 3e-05 |  |
|  | 2oam.A | 1kft.A |  |  |  |  | 30 | 4e-06 |  |
| * | 2opt.A | 2zcm.A | PF00440 |  | 3677 |  | 32 | 2e-06 |  |
| * | 2ove.A | 1kt6.A | PF00061 |  | 5576 | DAO.REA.0.636364 | 32 | 1e-06 |  |
|  | 2paw.A | 1y0n.A |  |  |  |  | 29 | 7e-06 |  |
| * | 2pfk.A | 3ff1.B |  |  | 5737 |  | 32 | 2e-05 |  |
| * | 2pkf.A | 3iq0.A | PF00294 |  | 166 | ACP.ATP.0.9375 | 32 | 9e-06 |  |
|  | 2q5r.A | 1s4x.A |  |  |  |  | 28 | 7e-06 |  |
|  | 2q6z.A | 1zan.L |  |  |  |  | 32 | 9e-06 |  |
|  | 2qev.A | 1s3q.G |  |  |  |  | 28 | 2e-05 |  |
|  | 2qsu.A | 1g6h.A |  |  |  |  | 31 | 1e-05 |  |
|  | 2qvl.A | 2l6e.A |  |  |  |  | 30 | 4e-06 |  |
| * | 2qys.A | 2ep5.B |  |  | 16491 |  | 32 | 1e-05 |  |
|  | 2r60.A | 2x4k.A |  |  |  |  | 29 | 7e-06 |  |
|  | 2rg7.A | 3tef.A |  |  |  |  | 31 | 1e-05 |  |
| * | 2rjd.A | 3oa6.B |  |  | 5634 |  | 29 | 2e-05 |  |
|  | 2sga.A | 2hbt.A |  |  |  |  | 29 | 2e-05 |  |
| * | 2sil.A | 3ibm.B |  |  |  | K.K.1 | 29 | 5e-05 |  |
| * | 2ts1.A | 3hv0.B | PF00579 |  | 166 |  | 32 | 2e-05 |  |
|  | 2uyo.A | 4i1k.B |  |  |  |  | 28 | 4e-05 |  |
|  | 2v78.A | 2i7r.A |  |  |  |  | 28 | 4e-05 |  |
|  | 2vfb.A | 3rgh.B |  |  |  |  | 26 | 1e-04 |  |
|  | 2vfy.A | 1te5.B |  |  |  |  | 31 | 7e-06 |  |
|  | 2vq4.A | 2dm4.A |  |  |  |  | 26 | 3e-05 |  |
|  | 2vua.A | 1kft.A |  |  |  |  | 30 | 4e-06 |  |
|  | 2wn4.A | 1dys.B |  |  |  |  | 32 | 2e-05 |  |
| * | 2wvh.A | 1kmq.A |  |  |  | MG.MG.1 | 31 | 1e-05 |  |
|  | 2wzt.A | 1ui0.A |  |  |  |  | 31 | 1e-05 |  |
| * | 2x5s.A | 1vpa.A |  |  | 16740 | GTP.CTP.0.742857 | 32 | 6e-06 |  |
|  | 2ywb.A | 1oi4.B |  |  |  |  | 32 | 1e-05 |  |
|  | 2ywm.A | 2k8s.A |  |  |  |  | 32 | 5e-07 |  |
| * | 2yxf.A | 1i1c.B | PF07654 |  |  |  | 32 | 5e-07 |  |
| * | 2yya.A | 1wr2.A |  |  | 3824 |  | 32 | 1e-05 |  |
| * | 2yyt.A | 3gdt.C | PF00215 | 4.1.1.23 | 3824 | C5P.BMP.0.869565 | 32 | 8e-06 |  |
| * | 2yzg.A | 2vpq.B |  |  | 3824 | ADP.ANP.0.8125 | 31 | 3e-05 |  |
|  | 2zbs.A | 2xrh.A |  |  |  |  | 28 | 1e-05 |  |
| * | 2zcg.A | 4dbe.B |  | 4.1.1.23 | 3824 | OMP.BMP.0.84 | 32 | 8e-06 |  |
|  | 2zco.A | 2kzh.A |  |  |  |  | 29 | 2e-05 |  |
| * | 2zgl.A | 2wt1.A | PF00337 |  | 30246 | A2G.BGC.0.6875 | 30 | 1e-05 |  |
|  | 2zhy.B | 1br9.A |  |  |  |  | 30 | 1e-05 |  |
| * | 2zj8.A | 2duy.A | PF00633 |  | 3677 |  | 31 | 2e-06 |  |
| * | 3a0y.A | 1ysr.A | PF02518 |  | 5524 |  | 32 | 1e-06 |  |
|  | 3a2r.X | 2kt5.A |  |  |  |  | 30 | 8e-06 |  |
|  | 3a5q.A | 2kxj.A |  |  |  |  | 28 | 1e-05 |  |
|  | 3aap.A | 3r0h.E |  |  |  |  | 29 | 6e-05 |  |
| * | 3ado.A | 3ctv.A | PF00725 |  | 3857 |  | 30 | 8e-06 |  |
| * | 3app.A | 1b5f.B | PF00026 |  | 4190 |  | 31 | 1e-06 |  |
| * | 3b3g.A | 1l3i.C |  |  | 6479 | SAH.SAH.1 | 32 | 4e-06 |  |
|  | 3ba1.A | 3va4.B |  |  |  |  | 31 | 6e-06 |  |
| * | 3blm.A | 3l86.A |  |  |  | CEM.NLG.0.6875 | 28 | 9e-05 |  |
| * | 3btv.A | 3ec7.A | PF01408 |  | 16491 | NAD.NAD.1 | 32 | 1e-05 |  |
|  | 3bue.A | 2qpq.A |  |  |  |  | 29 | 7e-06 |  |
|  | 3byl.A | 2dj3.A |  |  |  |  | 29 | 3e-05 |  |
|  | 3c2e.A | 1yd8.H |  |  |  |  | 27 | 3e-05 |  |
|  | 3c8n.A | 2lo4.A |  |  |  |  | 27 | 9e-06 |  |
| * | 3caf.A | 1wwb.X | PF07679 |  |  |  | 32 | 2e-07 |  |
| * | 3cb6.A | 2v3z.A | PF00557 |  | 9987 |  | 32 | 2e-05 |  |
|  | 3cgz.A | 2hrb.A |  |  |  |  | 29 | 3e-05 |  |
|  | 3cou.A | 2af0.A |  |  |  |  | 30 | 5e-06 |  |
|  | 3cq1.A | 3k69.A |  |  |  |  | 28 | 9e-06 |  |
| * | 3crm.A | 4e22.A |  |  | 166 |  | 32 | 6e-06 |  |
| * | 3csr.A | 2xfd.A |  |  |  | NAG.GAL.0.6875 | 26 | 6e-05 |  |
|  | 3ctb.A | 2o14.A |  |  |  |  | 32 | 2e-05 |  |
| * | 3d0o.A | 3g5j.B |  |  |  | PYR.GOL.0.714286 | 30 | 7e-06 |  |
|  | 3d95.A | 2a5m.A |  |  |  |  | 28 | 2e-05 |  |
|  | 3drd.A | 1kmq.A |  |  |  |  | 30 | 4e-05 |  |
| * | 3dre.A | 4dio.B |  |  | 166 |  | 30 | 8e-05 |  |
| * | 3dul.A | 3dlc.A |  |  | 8168 | SAH.SAM.0.962963 | 31 | 6e-06 |  |
|  | 3e1s.A | 3c70.A |  |  |  |  | 29 | 1e-04 |  |
| * | 3e5b.A | 2hjp.A | PF00463 |  | 3824 |  | 32 | 1e-05 |  |
|  | 3eiz.A | 3m1x.A |  |  |  |  | 32 | 9e-07 |  |
| * | 3ek6.A | 3c1m.D | PF00696 |  | 166 | GTP.ADP.0.787879 | 32 | 9e-06 |  |
|  | 3etf.A | 1qlo.A |  |  |  |  | 25 | 4e-05 |  |
| * | 3evh.A | 1qgi.A |  |  |  | BEM.GCS.0.785714 | 32 | 1e-05 |  |
|  | 3ex9.A | 2lmc.A |  |  |  |  | 31 | 8e-07 |  |
| * | 3exr.A | 2czd.B | PF00215 |  | 3824 |  | 30 | 2e-05 |  |
| * | 3f1l.A | 2dkn.B |  |  | 16491 | NAP.NAI.0.916667 | 32 | 7e-06 |  |
| * | 3f6f.A | 3lxz.C | PF00043 |  | 16740 | GTT.GTT.1 | 32 | 2e-06 |  |
|  | 3ftd.A | 1ix5.A |  |  |  |  | 27 | 8e-05 |  |
| * | 3fv6.A | 4gqw.A |  |  | 30554 |  | 32 | 1e-06 |  |
| * | 3g1s.A | 3v75.A |  | 4.1.1.23 | 3824 |  | 32 | 5e-06 |  |
|  | 3gbt.A | 2gv0.A |  |  |  |  | 28 | 6e-05 |  |
|  | 3gd0.A | 2cw5.C |  |  |  |  | 30 | 6e-05 |  |
| * | 3gdk.A | 3ru6.B | PF00215 | 4.1.1.23 | 3824 |  | 31 | 2e-05 |  |
|  | 3glk.A | 4ir8.B |  |  |  |  | 32 | 2e-05 |  |
| * | 3gpg.A | 3eti.F | PF01661 |  |  | APR.APR.1 | 29 | 1e-05 |  |
|  | 3gqh.A | 3tk9.A |  |  |  |  | 30 | 6e-06 |  |
|  | 3gsz.A | 3qvp.A |  |  |  |  | 32 | 6e-05 |  |
| * | 3gva.A | 1sg4.A |  |  | 3824 |  | 30 | 5e-06 |  |
|  | 3h2g.A | 1n1d.A |  |  |  |  | 30 | 1e-05 |  |
| * | 3h38.A | 1nrj.B |  |  |  | ATP.GTP.0.909091 | 30 | 4e-05 |  |
| * | 3h49.A | 2ajr.B | PF00294 |  | 16301 |  | 32 | 2e-05 |  |
|  | 3h71.A | 2okq.A |  |  |  |  | 30 | 2e-05 |  |
|  | 3hbh.A | 1tk7.A |  |  |  |  | 25 | 7e-05 |  |
|  | 3his.A | 2b0g.A |  |  |  |  | 25 | 1e-04 |  |
|  | 3hj4.A | 3us4.A |  |  |  |  | 29 | 1e-05 |  |
|  | 3hnx.A | 1x6m.B |  |  |  |  | 25 | 2e-04 |  |
|  | 3i0c.A | 3bkw.B |  |  |  |  | 30 | 3e-05 |  |
| * | 3i3i.A | 2wg9.B |  |  | 4623 |  | 27 | 2e-05 |  |
| * | 3i8s.A | 1puj.A |  |  | 5525 | GCP.GNP.0.939394 | 32 | 7e-06 |  |
|  | 3ik8.A | 2z3y.A |  |  |  |  | 30 | 1e-05 |  |
| * | 3ily.A | 1jl3.A | PF01451 |  | 4725 |  | 32 | 7e-07 |  |
|  | 3ity.A | 1qrj.A |  |  |  |  | 30 | 3e-05 |  |
|  | 3iuj.A | 1y0h.A |  |  |  |  | 28 | 4e-05 |  |
| * | 3jyl.A | 1uuf.A | PF00107 |  | 8270 |  | 31 | 3e-05 |  |
|  | 3k0m.A | 4dzb.B |  |  |  |  | 32 | 3e-06 |  |
|  | 3k5o.A | 3mab.A |  |  |  |  | 31 | 6e-06 |  |
| * | 3kaj.A | 2y4r.A |  |  |  | SO4.SO4.1 | 32 | 2e-05 |  |
|  | 3kdh.A | 2flh.C |  |  |  |  | 32 | 1e-06 |  |
| * | 3kje.A | 1hdg.Q |  |  |  | ADP.NAD.0.613636 | 30 | 3e-05 |  |
|  | 3kjt.A | 2gz5.A |  |  |  |  | 32 | 2e-05 |  |
| * | 3kp7.A | 3deu.B |  |  | 3700 |  | 30 | 7e-06 |  |
|  | 3kr9.A | 3uh0.A |  |  |  |  | 30 | 5e-05 |  |
|  | 3kx7.A | 2kcw.A |  |  |  |  | 32 | 7e-07 |  |
| * | 3lig.A | 1y4w.A | PF00251 |  | 4553 |  | 32 | 7e-05 |  |
|  | 3loi.A | 3ikb.B |  |  |  |  | 28 | 6e-05 |  |
|  | 3m4d.A | 1yg6.D |  |  |  |  | 29 | 5e-05 |  |
| * | 3n6j.A | 3dip.B |  |  | 3824 |  | 32 | 2e-05 |  |
| * | 3nk6.A | 1mxi.A | PF00588 |  | 1510 | SAM.SAH.0.962963 | 30 | 1e-05 |  |
| * | 3pte.A | 1ul3.B |  |  |  | GOL.GOL.1 | 27 | 6e-05 |  |
| * | 4ake.A | 3iij.A |  | 2.7.4.3 | 166 | ADP.ADP.1 | 31 | 5e-06 |  |
| * | 4pgm.A | 3eoz.A | PF00300 |  |  |  | 32 | 4e-06 |  |
| * | 4pti.A | 2kcr.A |  |  | 4867 |  | 31 | 4e-08 |  |

# References

1. Miller BT, Singh RP, Klauda JB, Hodoscek M, Brooks BR, et al. (2008) CHARMMing: a new, flexible web portal for CHARMM. J Chem Inf Model 48: 1920-1929.

2. Ye Y, Godzik A (2003) Flexible structure alignment by chaining aligned fragment pairs allowing twists. Bioinformatics 19 (suppl 2): ii246-ii255.

3. Chen YC, Wright JD, Lim C (2012) DR_bind: a web server for predicting DNA-binding residues from the protein structure based on electrostatics, evolution and geometry. Nucleic Acids Res 40: W249-W256.

4. Dessailly BH, Lensink MF, Orengo CA, Wodak SJ (2008) LigASite--a database of biologically relevant binding sites in proteins with known apo-structures. Nucleic Acids Res 36: D667-D673.

5. Altschul SF, Gish W, Miller W, Myers EW, Lipman DJ (1990) Basic local alignment search tool. J Mol Biol 215: 403-410.

6. Velankar S, Dana JM, Jacobsen J, van Ginkel G, Gane PJ, et al. (2013) SIFTS: Structure Integration with Function, Taxonomy and Sequences resource. Nucleic Acids Res 41: D483-D489.

1. *

   To whom correspondence should be addressed. [↑](#footnote-ref-2)
